# Phase separation modulates the assembly and dynamics of a polarity-related scaffold-signaling hub

**DOI:** 10.1038/s41467-022-35000-2

**Published:** 2022-11-23

**Authors:** Wei Tan, Sihua Cheng, Yingying Li, Xiao-Yang Li, Ning Lu, Jingxian Sun, Guiyue Tang, Yujiao Yang, Kezhu Cai, Xuefei Li, Xijun Ou, Xiang Gao, Guo-Ping Zhao, W. Seth Childers, Wei Zhao

**Affiliations:** 1 CAS Key Laboratory of Quantitative Engineering Biology, Shenzhen Institute of Synthetic Biology, Shenzhen Institutes of Advanced Technology, Chinese Academy of Sciences, Shenzhen, 518055 China; 2 Department of Pharmacy, School of Life Sciences, Henan University, Kaifeng, 475004 China; 3 CAS Key Laboratory of Synthetic Biology, CAS Center for Excellence in Molecular Plant Sciences, Shanghai Institute of Plant Physiology and Ecology, Chinese Academy of Sciences, Shanghai, 200032 China; 4 University of Chinese Academy of Sciences, Beijing, 100049 China; 5 Department of Materials Science and Engineering, School of Engineering, Southern University of Science and Technology, Shenzhen, 518055 China; 6 Department of Biology, School of Life Sciences, Southern University of Science and Technology, Shenzhen, 518055 China; 7 State Key Lab of Genetic Engineering & Institutes of Biomedical Sciences, Department of Microbiology and Microbial Engineering, School of Life Sciences, Fudan University, Shanghai, 200433 China; 8 Department of Chemistry, University of Pittsburgh, Pittsburgh, PA 15260 USA

**Keywords:** Intrinsically disordered proteins, Cell-cycle proteins, Apicobasal polarity, Differentiation, Bacterial development

## Abstract

Asymmetric cell division (ACD) produces morphologically and behaviorally distinct cells and is the primary way to generate cell diversity. In the model bacterium *Caulobacter crescentus*, the polarization of distinct scaffold-signaling hubs at the swarmer and stalked cell poles constitutes the basis of ACD. However, mechanisms involved in the formation of these hubs remain elusive. Here, we show that a swarmer-cell-pole scaffold, PodJ, forms biomolecular condensates both in vitro and in living cells via phase separation. The coiled-coil 4–6 and the intrinsically disordered regions are the primary domains that contribute to biomolecular condensate generation and signaling protein recruitment in PodJ. Moreover, a negative regulation of PodJ phase separation by the stalked-cell-pole scaffold protein SpmX is revealed. SpmX impedes PodJ cell-pole accumulation and affects its recruitment ability. Together, by modulating the assembly and dynamics of scaffold-signaling hubs, phase separation may serve as a general biophysical mechanism that underlies the regulation of ACD in bacteria and other organisms.

## Abstract (web summary)

The polarization of distinct scaffold-signaling hubs at opposite cell poles constitutes the basis of asymmetric cell division. Here, the authors show that phase separation serves as a general mechanism to regulate the assembly and dynamics of a new-pole scaffold-signaling hub.

## Introduction

**Fig. 1:**
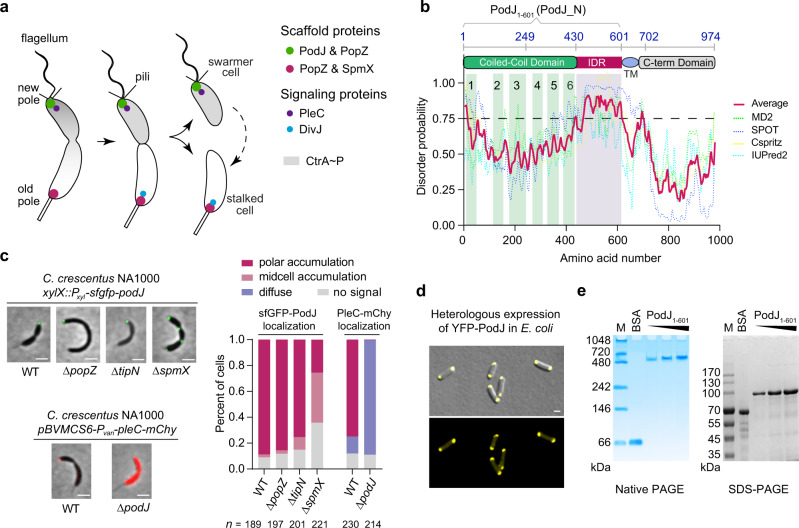
The asymmetrically localized PodJ is a self-assembled protein. **a** Schematic of asymmetric cell division of *C. crescentus*. The scaffolds PodJ-PopZ at the swarmer cell pole and PopZ-SpmX at the stalked cell pole recruit distinct signaling proteins, such as PleC and DivJ, respectively. After cell division, the swarmer cells develop into a stalked cell, which is correlated with the remodeling of the cell pole from a PodJ-rich signaling hub to an SpmX-rich signaling hub. **b** Detection of an intrinsically disordered region (IDR) in PodJ. The probability of IDR is represented as average scores (red line) calculated by Metadisorder MD2^59^, SPOT^60^, Cspritz^61^, and IUPred2^62^. Domain prediction was adapted from previous studies^15, 19^. TM transmembrane domain. **c** PodJ polar accumulation is independent of PopZ and TipN, but is affected by SpmX in *C. crescentus*. As a control, PleC accumulation was dependent on PodJ. A quantitative analysis of PodJ accumulation is shown on the right panel. **d** Heterologous expression of YFP-PodJ in *E. coli* indicates that PodJ accumulation is independent of polarity proteins. **e** Purified PodJ_1-601_ oligomerizes into a huge complex as illustrated by the native PAGE and compared with that of the SDS-PAGE analysis. Bovine serum albumin (BSA) was used as a control. M, protein marker. All scale bars, 1 μm. Source data are provided in the Source Data file.

By polarizing different cell fate determinants at opposite cell poles, asymmetric cell division (ACD) that produces distinct daughter cells is an evolutionarily conserved mechanism to generate cell diversity in both eukaryotes and prokaryotes^1,2^. As a well-established model to study bacterial ACD^3–5^, *Caulobacter crescentus* produces a motile swarmer cell and a sessile stalked cell during each cell cycle (Fig. 1a). In the pre-division cell stage, the polar localization of two distinct membraneless signaling complexes, particularly the phosphatase PleC and the kinase DivJ, coordinates to modulate the phosphorylation levels of a set of downstream signaling proteins (including the master regulator CtrA) and determinate the cell fate of *C. crescentus*^6^.

Scaffold proteins are known to physically tether client proteins to specific cellular areas, functioning in spatial regulation of biological processes including signaling transduction, cytokinesis, morphogenesis, and ACD^7^. In *Drosophila* and *Caenorhabditis*, the asymmetric localization of signaling proteins such as αPKC or PKC3 by the Par scaffold system is essential for proper embryonic development^8,9^. In *C. crescentus*, the kinase DivJ is recruited to the stalked cell pole (old cell pole) through a PopZ-SpmX-DivJ sandwich^10^, while the phosphatase PleC is localized to the swarmer cell pole (new cell pole) by the PodJ scaffold^11–13^ (Fig. 1a).

PodJ was first identified as a polar organelle development protein via genetic mutation analysis^14^ and later characterized as the localization factor for multiple new-pole client proteins, such as PleC^13,15^, PopA^16^, CpaE/C^12^, and HfaA/B/D^17^. The inactivation of *podJ* results in defective chemotaxis and resistance to bacteriophage^11,18^. Moreover, hyperexpression of PodJ causes immediate cell division arrest, followed by filamentation and finally cell death^18^. Functional domain dissection^19^ suggests that PodJ contains a cytoplasmic N-terminus composed of a coiled-coil-rich region (CC1-6) followed by an unknown structured region, and a C-terminus passing through the membrane into the periplasm (Fig. 1b). The periplasmic region is comprised of a tetrapeptide co-repeat domain and a peptidoglycan binding domain that modulates the pili biogenesis and has been shown to be dispensable for PodJ localization at the cell pole. In addition, the full-length PodJ is regularly proteolyzed into a shortened cytoplasmic form during cell-cycle coordination of downstream signals^12,19,20^. Nevertheless, the biomolecular basis and regulatory mechanism underlying the PodJ-centered scaffold-signaling hub remain unclear.

Liquid–liquid phase separation (LLPS) driving the assembly of membraneless compartments is regarded as a general mechanism that is involved in regulating gene expression, signal transduction, stress responses, and age-related disorders^21–23^. The assemblies of eukaryotic nucleoli, centrosomes, RNA-enriched granules, and Alzheimer-related neurofibrillary tangles were shown to be mediated by LLPS^24–27^. In contrast, fewer studies of phase separation regulation have been reported in prokaryotes. Recent progress has revealed that LLPS is also involved in the organization of bacterial membraneless organelles, including nucleoid, RNP-bodies, and ACD complexes^28–32^. In the current study, we found that LLPS plays an essential role in the *C. crescentus* PodJ-signaling hub assembly. Both the coiled-coil 4–6 region (CC4–6) and the intrinsically disordered region (IDR) in PodJ functionally contributed to generating biomolecular condensates and forming the PodJ-client complexes. Moreover, a negative regulation of PodJ phase separation by the old-cell-pole scaffold protein SpmX was observed. SpmX inhibited PodJ condensate formation and impeded its cell-pole accumulation and client recruitment. Therefore, our findings revealed a biophysical mechanism that involves the scaffold-signaling hub assembly and dynamics and may contribute to ACD in *C. crescentus*.

## Results

### The new-cell-pole PodJ is a self-assembled protein

Previous studies based on immunofluorescence microscopy analysis have suggested that PodJ asymmetrically localizes at the new cell pole of *C. crescentus*^11,12^. We confirmed this localization pattern of PodJ in the present study using time-lapse microscopy in live cells: sfGFP-PodJ specifically accumulated at the new cell poles of pre-division and swarmer cells; When the swarmer cells transitioned into stalked cells, the sfGFP-PodJ signals at the old cell poles diminished and new sfGFP-PodJ foci became evident at the new cell poles (Supplementary Fig. 1a).

Live-cell imaging showed that PodJ polar accumulation occurred independently of other scaffold proteins, including PopZ^33,34^ and TipN^35^ (Fig. 1c). Moreover, PodJ formed foci when heterologously expressed in *Escherichia coli* cells (Fig. 1d). Note that the symmetric *E. coli* is evolutionarily divergent from *C. crescentus* and does not contain any homologs of known *C. crescentus* polarity proteins^36^. These observations indicate that PodJ is likely self-assembled in cells. To support this, we purified the soluble cytoplasmic portion of PodJ (PodJ_1-601_), which also accumulated in *E. coli* (Supplementary Fig. 1b) and *C. crescentus* (Supplementary Fig. 1c), and characterized its oligomeric state via native polyacrylamide gel electrophoresis (PAGE) analysis. Similar to the well-studied PopZ^33^, PodJ_1-601_ oligomerized into a huge complex of more than 480 kDa (~8-mer) (Fig. 1e), suggesting that PodJ is a self-assembled protein.

### PodJ forms biomolecular condensates both in vitro and in vivo

**Fig. 2:**
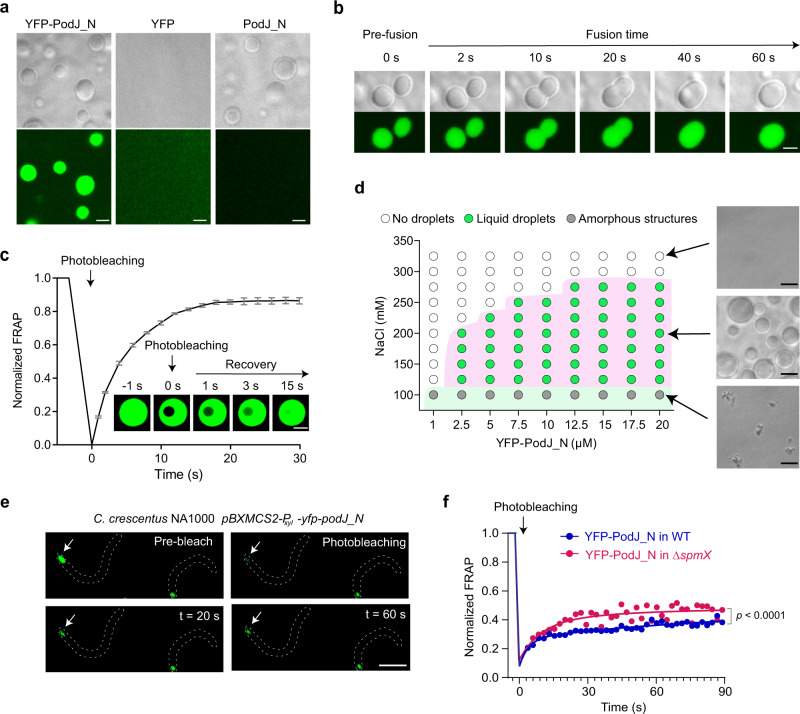
PodJ undergoes phase separation both in vitro and in *C. crescentus*. **a** The YFP-PodJ_N protein forms liquid droplets in vitro. Images were taken within 15 min after loading the ice-bathed proteins (5 µM) on a glass pad at 25 °C. YFP was used as a negative control. **b** The instantaneously contacted YFP-PodJ_N droplets tend to fuse together within 1 min. **c** FRAP analysis suggests that YFP-PodJ_N droplets are highly dynamic. The recovery curve was generated by averaging the signals of YFP-PodJ_N droplets (*n* = 6) from three independent experiments. One representative droplet is shown. The fluorescence intensity of pre-bleached droplets was normalized as 100%. Data are means ± SEM. **d** A regime diagram illustrates the formation of YFP-PodJ_N droplets at different protein and salt concentrations. Representative images are shown on the right panel. **e** FRAP analysis of YFP-PodJ_N condensates in *C. crescentus* wild-type and Δ*spmX* cells. The expression of YFP-PodJ_N was driven by the P_*xylX*_ promoter from a high copy plasmid to achieve FRAP analysis. One representative bleached focus (white arrow) in the wild-type cell is shown. **f** Quantification of the FRAP analyses in panel **e**. The recovery curves for each sample were generated by averaging the signals of YFP-PodJ_N foci (*n* = 6) from three independent experiments with nonlinear regression. The fluorescence intensity of pre-bleached foci was normalized as 100%. Data are means ± SEM and *p* value was determined by two-tailed Welch’s unpaired *t*-test. All scale bars, 2 μm. Source data are provided in the Source Data file.

To understand how oligomeric PodJ assembles at the polar hub, we tested possible phase separation after discovering a highly charged IDR^37,38^ and three tandem repeats (TRs) at the cytoplasmic terminus of PodJ (Fig. 1b and Supplementary Fig. 2a, b). In most cases, the presence of IDRs and TRs is closely linked with protein phase separation due to the generation of multivalent weak interactions^22^. Therefore, we performed in vitro LLPS experiments based on heterologous expression and purification of YFP-PodJ_1-601_ (hereafter designated as YFP-PodJ_N). YFP-PodJ_N formed micrometer-sized spherical droplets in a reconstituted buffer, which were clearly visible under both optical and fluorescence microscopies within 15 min of plating at 25 °C. In contrast, YFP alone produced dispersed fluorescence and no liquid droplets were observed (Fig. 2a). These results indicate that phase separation could be involved in PodJ_N assembly in vitro. Supporting this, time-lapse microscopy revealed that the instantaneously contacted YFP-PodJ_N liquid droplets tended to fuse into larger-sized droplets (Fig. 2b and Supplementary Video 1). Moreover, fluorescence recovery after photobleaching (FRAP) analysis demonstrated that the fluorescence intensity of the bleached YFP-PodJ_N droplets could be recovered within seconds (~85% recovery within ~15 s) (Fig. 2c and Supplementary Video 2). Therefore, these results indicate that PodJ_N droplets have liquid-like properties with high fluidity and high dynamics.

We next examined the factors that were previously shown to affect in vitro LLPS, including protein concentration, salt concentration, and temperature^22,27^. YFP-PodJ_N formed liquid-like droplets at protein concentrations as low as 2.5 µM (Fig. 2d). However, amorphous and irreversible structures were observed when the salt concentration was lower than 100 mM NaCl (Fig. 2d and Supplementary Fig. 2c). YFP-PodJ_N droplets emerged more quickly if the assay was executed at higher temperatures. Moreover, disassembly of the droplets was observed with time, and the higher the temperature, the faster disassembly was detected (Supplementary Fig. 2d, e). Collectively, these observations indicate that the YFP-PodJ_N droplets are unstable and could be manipulated in vitro.

A previous study has suggested that the absolute number of PodJ protein molecules per *C. crescentus* cell is ~1700^39^. Based on this, we estimated that the concentration of PodJ was approximately 0.8 mM at the *Caulobacter* cell pole (see Methods). This protein concentration is approximately 300-fold higher than the minimum concentration at which PodJ LLPS occurs in vitro (Fig. 2d), indicating that it could be sufficient to trigger PodJ condensate formation in vivo. Indeed, transmission electron microscopy (TEM) of *C. crescentus* cells with the overexpression of PodJ revealed that the new cell pole was packed with electron-dense protein (Supplementary Fig. 3a). Similar cell compartments were also observed in *E. coli* cells over-expressing either PodJ or PodJ_N (Supplementary Fig. 3b). Moreover, FRAP analyses in *C. crescentus* (Fig. 2e, f) and in *E. coli* (Supplementary Fig. 4a, b) suggested that YFP-PodJ_N molecules were constantly exchanged between the condensates and the surrounding aqueous solution. This process happened in tens of seconds (Fig. 2e, f), which is much faster than the possible disassemble/reassemble events and is consistent with the liquid-like properties^21^. Hence, these results confirm that PodJ overexpression leads to the formation of biomolecular condensates in vivo.

However, slower recovery rates for the in vivo samples (Fig. 2e, f and Supplementary Fig. 4a, b) were noticed when comparing with those of the in vitro samples (Fig. 2c), indicating that some important components may affect PodJ fluidity in vivo. These components could include the protein regulators, clients, temperature, and space-structures (e.g., crowding of the pole)^23,27,40,41^. The cell membrane to which PodJ is tethered may also affect the fluidity, since a significant decrease in the recovery rate was observed for the full-length PodJ when over-expressed in *E. coli* (compared to PodJ_N lacking the transmembrane domain and the C-terminus) (Supplementary Fig. 4b). Besides, experimental conditions and procedures could affect the FRAP results as well. For example, the whole-droplet FRAP may produce much lower recoveries than the partial-droplet FRAP as reported before^42,43^.

### Either CC4–6 or IDR is sufficient to drive PodJ LLPS in vitro

**Fig. 3:**
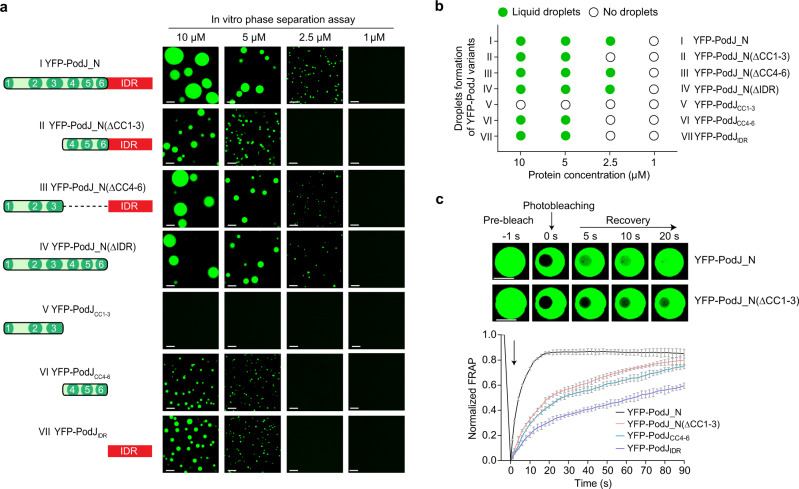
Either IDR or CC4–6 can mediate PodJ phase separation in vitro. **a** Droplet formation for the truncated YFP-PodJ_N proteins. Images were taken within 15 min after loading 1, 2.5, 5, or 10 µM YFP-PodJ_N and its variants on the glass pad at 25 °C. **b** Summary of droplet formation by YFP-PodJ_N and the variants in panel **a**. **c** Comparative FRAP analysis reveals a faster fluorescence recovery for YFP-PodJ_N droplets than for YFP-PodJ_N(ΔCC1–3) droplets. The recovery curves for each sample were generated by averaging the signals of YFP-PodJ_N droplets (*n* = 6) from three independent experiments, and the representative droplets are shown. The fluorescence intensity of the pre-bleached droplets was normalized as 100%. Data are means ± SEM. All scale bars, 5 μm. Source data are provided in the Source Data file.

Given that multiple domains were predicted in PodJ (Fig. 1b), we asked which domain is responsible for PodJ phase separation. A set of truncation variants based on PodJ_N were constructed and examined using standard assays for LLPS droplet formation (Fig. 3a). The YFP-PodJ variants, including YFP-PodJ_N(ΔCC1–3), YFP-PodJ_N(ΔCC4–6), and YFP-PodJ_N(ΔIDR), all formed liquid droplets in the reconstituted buffer (Fig. 3a, II–IV), whereas the protein variant simultaneously lacking the IDR and CC4–6 domains (YFP-PodJ_CC1–3_) did not exhibit droplet formation (Fig. 3a, V). These observations indicate that the PodJ LLPS is mediated by either the IDR or the CC4–6 domain in vitro. Supporting this, the PodJ variants with singular domains, i.e., YFP-PodJ_CC4–6_ (Fig. 3a, VI) and YFP-PodJ_IDR_ (Fig. 3a, VII), were able to form clear liquid droplets under the same assay conditions as above. To better understand the domain(s) responsible for PodJ LLPS in vivo, we expressed these variants in *E. coli* to observe their cellular accumulation and fluidity using FRAP analyses. Both YFP-PodJ_CC1–3_ and YFP-PodJ_CC4–6_ were able to form polar clusters while only YFP-PodJ_CC4–6_ displayed a visible fluidity in *E. coli*. YFP-PodJ_IDR_ was unable to form polar clusters and displayed no evident fluidity in *E. coli* (Supplementary Fig. 4a, b). Therefore, these results support the conclusion that either CC4–6 or IDR is sufficient to drive PodJ LLPS in vitro, while the in vivo PodJ LLPS may largely depend on CC4–6.

The capability to form polar clusters but lose fluidity of YFP-PodJ_CC1–3_ was interesting. Our previous work indicated that the CC1–3 domain is critical for PodJ_N to accumulate at the cell poles^13^. Indeed, YFP-PodJ_CC1–3_ can phase separate at a higher concentration with a C_sat_ of ~15 μM. This is a concentration about 3-fold higher than that of other domains including CC4–6 and IDR under the same assay conditions (Fig. 3b and Supplementary Fig. 5a). Therefore, the cellular accumulation of YFP-PodJ_CC1–3_ was investigated more closely using inclusion body detection assays in *E. coli*. In comparison to other variants, PodJ_CC1–3_ tended to exclude the soluble mCherry from the polar clusters (Supplementary Fig. 5b) and co-localized with the inclusion body marker IbpA^44^ (Supplementary Fig. 5c). These observations indicate that the cellular accumulation of YFP-PodJ_CC1–3_ may mainly form insoluble aggregates rather than liquid-like condensates. Consistent with this, an evident aging^45^ of YFP-PodJ_CC1–3_ droplets versus YFP-PodJ_N droplets was observed in vitro through FRAP analysis (Supplementary Fig. 5d).

Nevertheless, CC1–3 may function in adding the driving force of condensates by providing a certain level of rigidity in PodJ, similar to the observation in Gcn4-Med15 complex^46,47^. Consistent with this assumption, adding the CC1–3 domain back to either YFP-PodJ_CC4–6_ or YFP-PodJ_IDR_ resulted in the earlier emergence of droplets at the same protein concentrations (Fig. 3a, b). Moreover, FRAP analysis showed that fluorescence recovered more rapidly for YFP-PodJ_N droplets than for YFP-PodJ_N(ΔCC1–3), suggesting that CC1–3 increased the fluidity of the PodJ droplets in vitro (Fig. 3c). Collectively, these results indicate that the N-terminal CC1–3 functions in promotion of PodJ LLPS rather than phase separation itself.

### PodJ recruits client proteins via IDR and CC4–6

**Fig. 4:**
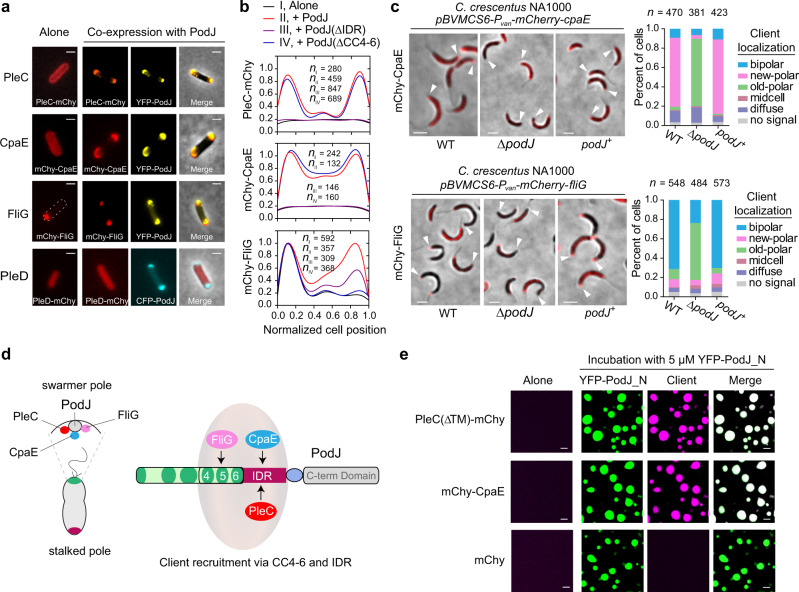
PodJ recruits client proteins via the LLPS-related domains. **a** Identification of PodJ clients in *E. coli*. Three out of 23 proteins (PleC, CpaE, and FliG) were identified by co-expressing them with PodJ to observe their subcellular localization changes in *E. coli*. PleD is shown as a negative control. **b** Quantitative analyses of the client signal along the cell lengths. These client proteins were expressed alone or co-expressed with PodJ (or PodJ variants) using the same induction concentration in *E. coli*. Data were normalized with the highest intensity as 100% in protein localized strains. **c** Confirmation of the recruitment for CpaE and FliG to the new cell poles by PodJ in *C. crescentus*. mCherry-CpaE and mCherry-FliG were expressed in wild-type (WT), Δ*podJ*, or *podJ* complementary strains (*podJ*^*+*^: *C. crescentus* Δ*podJ*, *xylX::P*_*xyl*_*-podJ*), respectively, to observe their subcellular localization changes. Quantifications of the client localization patterns are shown in the right panels. White arrows, new cell poles. **d** Schematic illustration of the client protein recruitment by PodJ. IDR is required for the recruitment of PleC and CpaE while CC4–6 is required for the recruitment of FliG. **e** PodJ recruits PleC and CpaE via LLPS in vitro. YFP-PodJ_N was incubated with 10 µM PleC(∆TM)-mCherry or mCherry-CpaE for 15 min before imaging. The mCherry was used as a negative control. All scale bars, 2 µm. Source data are provided in the Source Data file.

Previous studies have suggested that PodJ could serve as a scaffold for the recruitment of PleC^12^, PopA^16,48^, and CpaE^10,48^, among which, PleC and PopA were shown to be recruited directly by PodJ^13,16^. To further explore PodJ’s recruitment capability, we conducted co-localization experiments by examining 23 cell cycle- or polarity-related proteins from the *C. crescentus* localisome^49^ (Supplementary Table 1). In brief, these proteins were expressed alone or co-expressed with PodJ in *E. coli* and the possible changes in subcellular accumulation were monitored. This screen identified another two proteins, i.e., CpaE and FliG, which were directly recruited by PodJ to the *E. coli* cell poles (Fig. 4a, b). CpaE is a known pilus assembly protein that is required for polar pili biogenesis^35,50^, while FliG is a flagellar motor switch protein that functions as a component of the flagellar cytoplasmic ring and is essential for motor assembly^51^. The recruitment of these two proteins by PodJ was further confirmed by genetic analyses in *C. crescentus* (Fig. 4c) and time-lapse assays in *E. coli* (Supplementary Fig. 6).

Due to the one-to-many recruitment, PodJ-client interactions may not fully fit the classic protein-protein interaction mechanisms such as “Lock and Key” or “Induced Fit”. We sought to understand how PodJ interacted with diverse client proteins, and the first thing needed was to clarify the interacting domain(s) with these clients in PodJ. We conducted co-localization experiments by co-expressing the truncated PodJ proteins with clients in *E. coli*. Quantitative results showed that IDR was responsible for the recruitment of PleC and CpaE, while CC4–6 was responsible for the interaction with FliG (Fig. 4b and Supplementary Fig. 7). Hence, these results reveal a possible relationship between PodJ LLPS and the recruitment of client proteins, by targeting the same domains: IDR and CC4–6 (Fig. 4d).

In the current study, PleC and CpaE were selected and purified to perform the in vitro LLPS experiments in the presence or absence of PodJ. We noticed that neither PleC(∆TM)-mCherry nor mCherry-CpaE could form visible droplets alone, even at a protein concentration of 10 µM. However, when these proteins were co-incubated with 5 µM YFP-PodJ_N, clear liquid droplets were formed immediately (Fig. 4e). Taken together, these results suggest that PodJ recruits client proteins such as PleC and CpaE via the LLPS-related domains. Given the dynamic characteristics of biomolecular condensates, we speculate the versatile recruitment capability of PodJ could be derived from LLPS rather than the conserved conformational interactions^24^.

### SpmX inhibits PodJ LLPS and affects its client recruitment ability

**Fig. 5:**
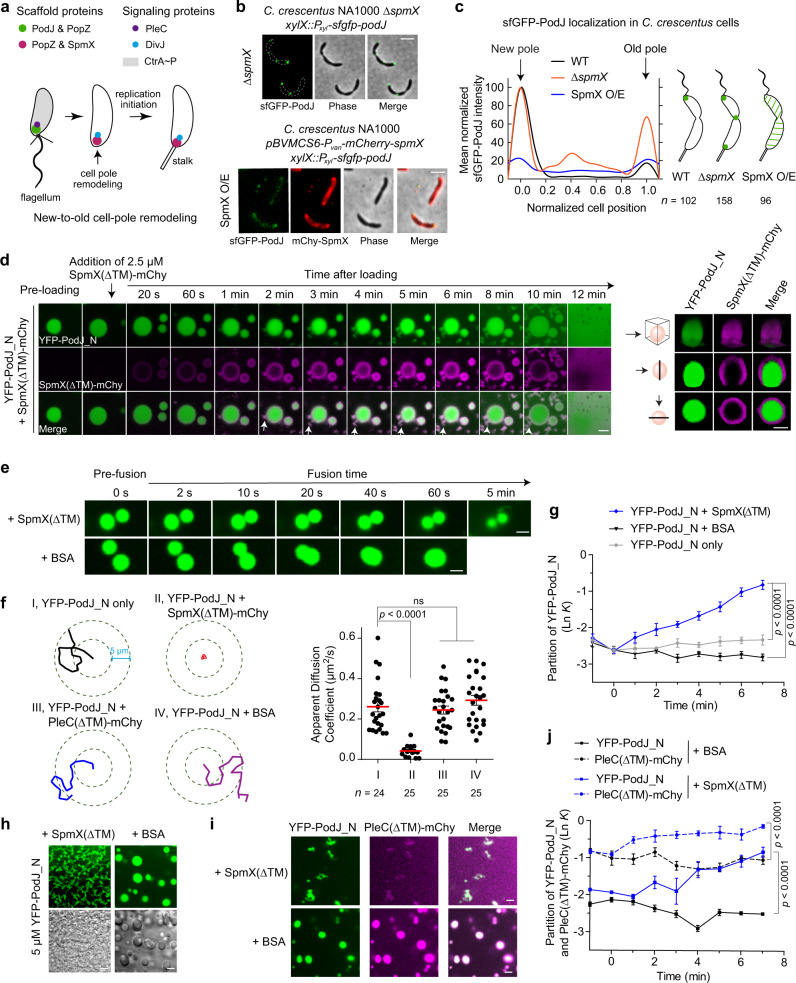
PodJ phase separation and client recruitment are regulated by SpmX. **a** Schematic of the new-to-old cell-pole remodeling in *C. crescentus*. Accompanied by the expression of SpmX, the swarmer cell pole is remodeled from a PodJ-PleC-rich hub to a SpmX-DivJ-rich hub. **b** PodJ subcellular localization is negatively regulated by SpmX in *C. crescentus*. Representative sfGFP-PodJ expressing cells induced by the same concentration of xylose are shown after deletion of *spmX* or overexpression (O/E) of SpmX for 3 h. **c** Quantitative analysis of sfGFP-PodJ signals in different *C. crescentus* strains. Data were normalized with the highest intensity as 100% in wild-type and ∆*spmX* strains. **d** Interfacial interaction between PodJ and SpmX. The solution of pre-formed YFP-PodJ_N (5 µM) droplets was mixed with 2.5 µM SpmX(∆TM)-mCherry. Three-dimensional photomicrographs are shown on the right panel (top, *y*-stack; middle, single *z-y* plane; lower, single *x-y* plane). White arrows, amorphous aggregates. **e** The adjacent YFP-PodJ_N droplets failed to fuse together when 2.5 µM SpmX(∆TM) was added. BSA (2.5 µM) was used as the negative control. **f** The YFP-PodJ_N droplets have very restricted trajectories in the presence of SpmX(∆TM)-mCherry. Representative trajectories and apparent diffusion coefficients of samples are shown on the left and right panels, respectively. **g** YFP-PodJ_N partitions less into the condensed phase after the addition of SpmX(∆TM) (*n* = 6). **h** YFP-PodJ_N results in no droplets when pre-incubated with SpmX(∆TM). BSA (10 µM) was used as the negative control. Images were acquired after 5 min of incubation. **i** The negative regulation of PodJ impedes the PleC partitioning by SpmX. 5 µM YFP-PodJ_N and 5 µM PleC(∆TM)-mCherry were incubated for 15 min. Then, 2.5 µM SpmX(∆TM) was added and images were acquired after another 5 min. The addition of 2.5 µM BSA was used as the control. **j** Partitioning analysis of YFP-PodJ_N (solid line) and PleC(∆TM)-mCherry (dashed line) in panel **i** (*n* = 6). Data are means ± SEM and *p* value was determined by one-way ANOVA (**f**) or two-tailed Welch’s unpaired *t*-test (**g** and **j**). ns, non-significant. All scale bars, 2 µm. Source data are provided in the Source Data file.

In *C. crescentus*, as the newborn swarmer cells transition into stalked cells, the inherited PodJ/PleC-rich signaling hub undergoes compositional remodeling to become an SpmX/DivJ-rich signaling hub^10^ (Fig. 5a). However, a key question remains: how is this new-to-old cell-pole remodeling achieved and regulated?

Eukaryotic studies have suggested that inhibitory regulation of interactions between the asymmetrically partitioned complexes is essential for the robust establishment of cell polarity^9^. Based upon this logic, we screened a set of 11 polarity proteins that reside at the cell poles from the *C. crescentus* localisome^49^ and examined their effects on PodJ subcellular localization in *E. coli* (Supplementary Table 2). Finally, an old-cell-pole scaffold protein SpmX was identified, the presence of which dramatically impeded the polar accumulation of PodJ in *E. coli* (Supplementary Fig. 8a). We confirmed that the subcellular accumulation of PodJ depends upon SpmX in *C. crescentus* (Fig. 5b, c). Deletion of *spmX* resulted in a long-chain cell phenotype in *C. crescentus*^52^, and each constriction site was occupied by a PodJ focus. The percentage of cells containing bipolar PodJ (including mid-cell PodJ) increased to ~90% in ∆*spmX* compared to that of the wild-type strain (Fig. 5c). In contrast, overexpression of SpmX resulted in a significant reduction of the PodJ signal at the cell poles (Fig. 5b, c). These results indicate that SpmX negatively regulates PodJ subcellular accumulation.

To understand the details of SpmX regulation upon PodJ, we designed an in vivo experiment to show the dynamic changes of PodJ condensates by inducing the cells with a single copy of *mCherry-spmX*. The titration with mCherry-SpmX using a higher concentration of inducer resulted in more dissociation of sfGFP-PodJ at the cell poles (especially at the old cell pole) of *C. crescentus* (Supplementary Fig. 8b–d). In contrast, no obvious decrease of sfGFP-PodJ signal was detected at the cell poles without induction of mCherry-SpmX. These results suggest that SpmX may function in the disassembly of PodJ condensates at the old cell pole. The adverse titration of mCherry-SpmX using different concentrations of sfGFP-PodJ inducer was also executed (Supplementary Fig. 9). A faster accumulation of sfGFP-PodJ at the new cell pole was observed, comparing with that at the old cell pole, possibly due to the negative regulation from mCherry-SpmX at the old cell pole. These data further support that the subcellular formation of PodJ condensates was modulated by SpmX in vivo.

A recent study reported that SpmX also has the capability to be phase separated^32^. Our results confirmed the generation of liquid droplets using 5 µM SpmX(∆TM)-mCherry under the same assay conditions as PodJ (Supplementary Fig. 10a). To dissect the molecular mechanism of SpmX regulation upon PodJ accumulation, the PodJ phase separation in the presence of SpmX was monitored. By adding 5 µM SpmX(∆TM)-mCherry, the pre-formed YFP-PodJ_N droplets lost fluidity in 20 s and produced amorphous structures in ~2 min (Fig. 5d and Supplementary Video 3). The YFP-PodJ_N droplets were unable to fuse (Fig. 5e) and grow (Supplementary Fig. 10b), and exhibited very restricted trajectories, with an average apparent diffusion coefficient of *D* = 0.04 µm^2^ s^−1^ after the addition of SpmX(∆TM)-mCherry, compared to 0.28 µm^2^ s^−1^ when SpmX was absent (Fig. 5f and Supplementary Fig. 10c). Hence, these data suggest an inhibition of PodJ phase separation by SpmX in vitro. Moreover, an obvious assembly of SpmX(∆TM)-mCherry on the surface of YFP-PodJ_N condensates was monitored (Fig. 5d and Supplementary Video 3). The observation was similar to a recent discovery of interfacial interaction between MEG-3 and PGL-3 droplets during zygote polarization^53^, indicating that SpmX(∆TM)-mCherry can similarly impede the exchange of molecules between the YFP-PodJ_N droplets and the surrounding aqueous solution. Supporting this finding, the partitioning analyses showed that YFP-PodJ_N partitioning into the condensed phase was suppressed by SpmX(ΔTM) (Fig. 5g and Supplementary Fig. 10d). Moreover, the acceleration of disassembly of YFP-PodJ_N droplets was observed in the presence of SpmX(∆TM) in vitro (Supplementary Fig. 10e). On the other hand, YFP-PodJ_N pre-incubated with SpmX(∆TM) produced none of the PodJ droplets, but rather amorphous structures (Fig. 5h and Supplementary Fig. 10f). As a control, the pre-incubation with BSA did not affect the formation of the PodJ droplets (Fig. 5h). Collectively, these results suggest that the inhibition of PodJ phase separation likely resulted from the impairment of PodJ condensate dynamics and stability by SpmX.

We next assessed whether inhibiting PodJ phase separation could affect its protein recruitment ability by performing in vitro LLPS experiments using the client PleC as an example. Pre-incubation with SpmX(∆TM) caused amorphous aggregation of both PleC(∆TM)-mCherry and YFP-PodJ_N, impeding the formation of PodJ-PleC droplets (Supplementary Fig. 11a). We further detected the possible impediment of PodJ to PleC recruitment using a post-loaded SpmX, which agrees with the physiological progression in vivo^54^. YFP-PodJ_N and PleC(∆TM)-mCherry formed co-localized droplets immediately after incubation, as displayed in Fig. 4e. However, the droplets lost their fluidity as soon as SpmX(∆TM) was added, and amorphous aggregates were observed after approximate 4 min (Fig. 5i and Supplementary Fig. 11b). Moreover, partitioning analyses revealed that PleC(∆TM)-mCherry partitioned into the condensed phase to a lesser degree after SpmX(∆TM) addition, suggesting that the inhibition of PodJ dynamics by SpmX may also impede PleC recruitment (Fig. 5j and Supplementary Fig. 11c). Taken together, these in vitro results, combined with in vivo observations, suggest that new-to-old cell-pole remodeling could be driven by the inhibition of PodJ phase separation and the following client recruitment via SpmX.

## Discussion

ACD of *C. crescentus* gives rise to surface-associated (stalked) cells and free-living (swarmer) cells, enabling this aquatic bacterium to survive in nutrient-poor environments. The scaffolds, together with signaling proteins, are packed as membraneless hubs at distinct cell poles and determine the *C. crescentus* cell fates^13,33,34^. In the current study, we present evidence that LLPS, a previously unrecognized mechanism, is involved in the regulation of the assembly and dynamics of the new-cell-pole PodJ-signaling hub.

While there are still controversies regarding the extent and scope of LLPS regulation^23^, it has emerged as a general mechanism that involves the assembly of membraneless compartments in both eukaryotes and prokaryotes^28,55^. Studies have shown that bacterial cells are well-organized rather than appearing as just “a bag of enzymes”^16,31^. The organelle-like membraneless structures, such as nucleoid and polarity hubs, have been sequentially revealed in recent years^28,31,56^. In this study, PodJ phase separation provides a mechanistic example of LLPS regulation in bacterial scaffold-signaling hub assembly, which is a common phenomenon during the development of cell polarity in most living beings.

**Fig. 6:**
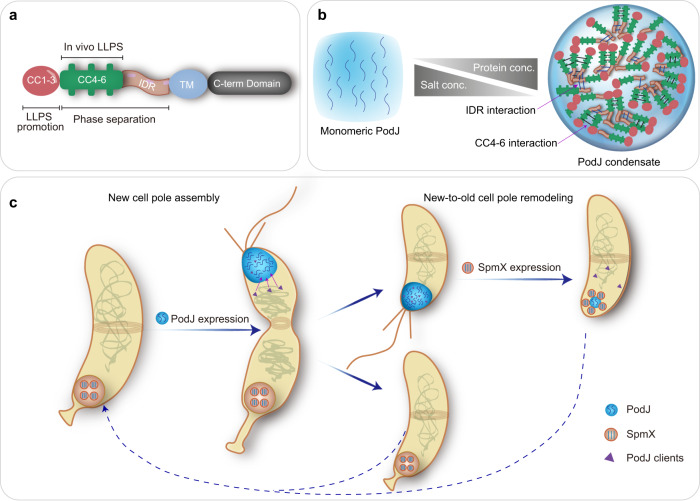
Schematic of PodJ phase separation and the proposed model for regulating bacterial asymmetric cell division. **a** Functional domain architecture of PodJ. Both CC4–6 and IDR are responsible for PodJ phase separation in vitro. CC4–6 is the sole domain sufficient to drive PodJ LLPS in vivo and CC1–3 is the domain functioning in LLPS promotion. **b** The monomeric PodJ_N undergoes phase separation into biomolecular condensates, which are regulated by the concentrations of protein and salt in vitro. **c** Phase separation is involved in the regulation of PodJ new cell pole assembly and new-to-old cell-pole remodeling. The assembly of scaffold-signaling complexes at the new cell pole is mediated by PodJ phase separation. In addition, the subcellular accumulation and the client recruitment of PodJ are negatively regulated by SpmX, which is also through the LLPS of the two proteins.

LLPS regulation of the PodJ-centered scaffold-signaling hub is shown in two ways (Fig. 6). First, LLPS is essential for the assembly and functionalization of the PodJ hub (Figs. 2 and 4). Both CC4–6 and IDR contribute to the phase separation and client recruitment capacity of PodJ (Figs. 3, 4, and 6a, b). On the other hand, CC1–3 may enhance the LLPS by providing a certain level of rigidity in PodJ^46,47^ (Figs. 3 and 6a and Supplementary Fig. 5). Deep analysis of CC4–6 using the RADAR algorithm^57^ has revealed three TRs characterized as AE#R#A#AI in the middle of each coiled-coil region (Supplementary Fig. 2b), which could be the cause of the CC4–6-mediated LLPS. These folded domains/multimeric units participating in multivalent interactions of phase-separated proteins have already been reported^40,41,58^. Second, LLPS is required for the modulation of PodJ dynamics through the negative regulator SpmX (Fig. 5 and Supplementary Figs. 8–10). The interfacial interaction between PodJ and SpmX was monitored in vitro. SpmX decreased the motion and accelerated the disassembly of PodJ condensates and impeded the following client partition, demonstrating a negative regulation of LLPS from a non-synthetic protein. These results, combined with the in vivo observations, suggest that the PodJ-SpmX interaction driven by LLPS could facilitate the new-to-old cell-pole remodeling in *C. crescentus* (Fig. 6c).

Nevertheless, it is still necessary to evaluate whether the protein concentrations and other factors used in in vitro LLPS experiments coincide with the physiological situation in *C. crescentus*. In the current study, the in vitro conditions that allow the formation of PodJ droplets were screened using a matrix of different concentrations of proteins and salts (Fig. 2d). The calculated cellular PodJ concentration met the minimal requirements for PodJ LLPS in vitro. The formation and dynamics of PodJ LLPS were also observed under physiological conditions both in vitro and in living cells (Figs. 2, 4, and 5). However, compared to that in vitro, an understanding of how phase separation regulation affects cellular PodJ function, including that in the periplasm, is still limited. Future work should focus on identifying key amino acid sites that determine PodJ phase separation and the physiological factors that regulate PodJ client recruitment via LLPS.

During cell pole remodeling, the disappearance of PodJ condensates at the old cell pole was accompanied by the expression of SpmX^10,54^ (Fig. 6c). In the present study, the connection between the old and new cell pole scaffolds was built by LLPS regulation. FRAP analysis showed that YFP-PodJ_N fluorescence recovered more quickly in Δ*spmX* than in wild-type cells (Fig. 2e), supporting the negative regulation of PodJ phase separation by SpmX in vivo. SpmX restricts the dynamics of PodJ condensates and causes less partitioning of PodJ into the condensed phase, which may lead to the dissociation of polar PodJ and the release of PodJ clients (Fig. 5 and Supplementary Figs. 8 and 11). However, a robust understanding of interaction sites between SpmX and PodJ is still lacking. We have constructed a series of PodJ truncations and executed the interaction experiments with SpmX(∆TM). The results indicate that the inhibition of PodJ LLPS by SpmX could result from the interfacial interaction with the CC4–6 domain. A surface assembly on YFP-PodJ_CC4–6_ droplets by SpmX(∆TM)-mCherry was reestablished in vitro and the dissociation of YFP-PodJ_CC4–6_ polar clusters in the presence of SpmX(∆TM) was demonstrated in *E. coli* (Supplementary Fig. 12). Moreover, possible interactions between PodJ_IDR_ and SpmX(∆TM) were also monitored and showed a different pattern from the interfacial binding (Supplementary Fig. 12). During the cell cycle of *C. crescentus*, SpmX expression is after the formation of PodJ condensates, implying that the interaction of these two scaffolds may be associated with the clearance of PodJ at the old cell pole^12^. Nevertheless, details of the multiple-domain interactions require further investigation. On the other hand, due to the difficulty in purifying the membrane-associated PodJ, we used the PodJ_N construct to mimic the full-length PodJ for most of our in vitro studies. However, some of these observations, such as the PodJ droplets matrix or the PodJ-SpmX interaction, may not fully represent the LLPS regulation or subcellular localization in cells. Future directions should aim to reconstitute the PodJ phase separation on supported lipid bilayers^23,32^ to mimic the membrane-bound topology or develop strategies to directly purify the full-length PodJ protein.

Taken together, this study presents a primary regulatory mechanism that is involved in bacterial new cell pole assembly and new-to-old cell-pole remodeling (Fig. 6c). The LLPS may serve as a general biophysical approach for assembling scaffold-signaling complexes and regulating ACD. Similar methods could be employed for the rational engineering of artificial organelles and other membraneless biocatalytic compartments.

## Methods

### Bacterial strains and growth conditions

All strains, plasmids, and oligonucleotides used in this study are listed in Supplementary Tables 3–5. Recombinant *C. crescentus* strains were obtained by the electroporation of integrating or replicating plasmids. *C. crescentus* cells were grown aerobically in peptone yeast extract (PYE) rich medium or M2G minimal medium containing 0.2% (w/v) glucose at 30 °C. Recombinant *E. coli* strains were obtained using the standard clone method and were grown aerobically in Luria–Bertani (LB) medium at 37 °C unless otherwise stated. The plasmids were constructed by Gibson assembly or the standard PCR-based mutagenesis method and were verified by DNA sequencing. All oligonucleotides were synthesized by Sangon Biotech.

When required, antibiotics were used at the following concentrations (liquid/solid media for *C. crescentus*; liquid/solid media for *E. coli*; µg ml^−1^): kanamycin (5/20; 50/50), chloramphenicol (1/2; 20/20), spectinomycin (not applicable; 50/50), ampicillin (not applicable; 100/100). All reagents used in this study were purchased from Sigma-Aldrich unless otherwise stated.

### Bioinformatic analyses of PodJ

The intrinsic disorder tendency of PodJ was analyzed using four independent programs: Metadisorder MD2^59^, SPOT^60^, Cspritz^61^, and IUPred2^62^. The scores of these programs were plotted against the PodJ sequence and assigned between 0 and 1, and a score above 0.5 indicates disorder. In the current study, an IDR was predicted with a disorder probability above 0.75 in PodJ.

TOPCONS^63^ and HHpred^64^ were used to analyze the protein topology and domain, respectively, especially in the prediction of transmembrane domains and the cellular orientations of proteins. Predicted cytoplasmic termini of these proteins were used for the construction of fluorescent fusion proteins.

Classification of Intrinsically Disordered Ensemble Regions (CIDER)^65^ was used to predict the charge distribution of PodJ_N.

The rapid automatic detection and alignment of repeats algorithm (RADAR)^57^ was used to detect the TRs in PodJ_N, and the results were manually cured.

### Protein expression and purification

The proteins purified and analyzed in this study are listed in Supplementary Table 6. Recombinant *E. coli* BL21(DE3) cells harboring the expression plasmid were grown in LB medium at 37 °C until the OD600 reached approximately 0.6, after which cells were induced with 0.5 mM Isopropyl β-D-1-thiogalactopyranoside (IPTG) at 16 °C for 12 h. Cell pellets were collected and resuspended in lysis buffer containing 25 mM HEPES (pH 7.5), 800 mM NaCl, 10% (v/v) glycerol, and 20 mM imidazole. After sonication and centrifugation, the supernatant was incubated with Ni^2+^-NTA agarose resin at 4 °C for 1 h, which was pre-equilibrated with the lysis buffer. After washing with 10–20 column volumes of wash buffer containing 25 mM HEPES (pH 7.5), 800 mM NaCl, 10% (v/v) glycerol, and 30 mM imidazole, the recombinant His-tagged protein was eluted from the agarose beads with elution buffer containing 25 mM HEPES (pH 7.5), 500 mM NaCl, 10% (v/v) glycerol, and 500 mM imidazole. When needed, the eluted proteins were concentrated with a dialysis bag (Sangon Biotech, SP132574, MWCO 10,000 g mol^−1^) immersed in PEG-8000 powder and further dialyzed in a dialysis buffer containing 25 mM HEPES (pH 7.5), 200 mM NaCl, 10% (v/v) glycerol, and 1 mM dithiothreitol (DTT) three times at 4 °C. Concentrations of purified proteins were determined using the Bradford protein assay kit (Beyotime Biotech, P0006). All recombinant proteins were obtained with purity >80% and their SDS-PAGE analyses are shown in Supplementary Fig. 13. The dialyzed proteins were stored at −80 °C before use.

### Native polyacrylamide gel electrophoresis (PAGE) analysis

To investigate the oligomeric state of PodJ, native PAGE analysis was performed. The purified PodJ_N protein was diluted using a 5× native sample loading buffer (Sangon Biotech, C506032) and separated on a 7.5% native PAGE gel (BBI, C601100). Bovine serum albumin (BSA) (BBI, A600903) was used as a control. The loading concentration of purified PodJ_N applied to each lane varied between 3.6 and 7.2 μg. The gel was run in 1× HEPES native PAGE running buffer (pH 7.5, BBI, C601110) at 80 V for 4 h at 4 °C, and stained with Coomassie brilliant blue R-250.

### In vitro liquid–liquid phase separation (LLPS) assay and data analysis

For the in vitro phase separation assay, all experiments were performed in a buffer containing 25 mM HEPES (pH 7.5), 10% (v/v) glycerol, 1 mM DTT, and 200 mM NaCl, unless otherwise stated. Approximately 2 µl (5 µM) purified protein solution (PodJ_N, YFP-PodJ_N, or SpmX(∆TM)-mCherry) was loaded onto a 35-mm glass bottom dish (Cellvis, D35-20-1-N) and imaged immediately using a Nikon A1R^+^ confocal laser scanning microscope equipped with a 100× oil immersion objective lens. The same amount of purified YFP or mCherry protein was used as the control. The specimens were illuminated with a 488-nm laser for yellow fluorescence and with a 561-nm laser for red fluorescence. YFP fluorescence was detected using the FITC filter (Nikon, excitation filter 480/15, dichroic mirror 505, and emission filter 535/20), and mCherry (mChy) fluorescence was detected using the TRITC filter (Nikon, excitation filter 540/25, dichroic mirror 565, and emission filter 605/55). All images were acquired using the same laser power, exposure time, gain, and offset settings at ~25 °C, unless otherwise stated.

Two types of sample loading methods were used to determine the effects of proteins (SpmX, PleC, or BSA) upon the PodJ phase separation. (I), 2 µl YFP-PodJ_N protein solution is loaded onto the glass bottom. When the YFP-PodJ_N droplets are formed (~15 min), another 2 µl of tested protein solution with indicated concentration is added onto the edge of the YFP-PodJ_N sample to let them touch each other, and the images are taken meanwhile. (II), 2 µl YFP-PodJ_N protein solution is mixed directly with 2 µl tested protein solution and loaded onto the glass bottom. The images are taken immediately.

The fluorescence intensity and the size of YFP-PodJ_N liquid droplets were quantitatively analyzed using MicrobeJ^66^. To visualize the interfacial interaction between PodJ and SpmX, the three-dimension (3D) image stacks (0.1 µm Z steps) were captured, and the mapping analysis was performed using 3Dscript^67^.

### Single-particle tracking and analysis

Single microspheres of YFP-PodJ_N droplets were tracked using the Fiji/ImageJ plugin MTrackJ. Microspheres on the glass surface or near the edge of the tested protein solutions were excluded from the data analysis to avoid artifacts. The feature size and minimum intensity of YFP-PodJ_N microspheres were empirically chosen so that most of the visible microspheres were detected (>80%) in a frame. For the tracks with at least 8 consecutive frames, their trajectory coordinates (*x*, *y*, *t*) were used to calculate the two-dimensional mean-squared displacement (MSD). The MSD(*t*) can be defined by Eq. (1):

1 $${{{{{\rm{MSD}}}}}}=\langle {|r(t)-r(0)|}^{2}\rangle$$

where *r*(*t*) is the position of the microspheres at time *t* and *r*(0) is the initial position.

The mean MSD was used to calculate the apparent diffusion coefficient (*D*) using Eq. (2):

2 $${{{{{\rm{MSD}}}}}}=4D{t}^{\alpha }$$

where *α* is the anomalous diffusion exponent obtained by linearly fitting the dataset with log(MSD) (*y*) and *t* (*x*). *D* is obtained by linearly fitting the dataset with MSD (*y*) and *t*^*α*^ (*x*). The MSD increases linearly with *t* for the PodJ_N droplets alone and the PodJ_N droplets in the presence of PleC(∆TM) or BSA (*α* ≈ 1), indicating that these PodJ_N microspheres did not experience subdiffusion. In contrast, the PodJ_N droplets in the presence of SpmX(∆TM) have a markedly different behavior with *α* ≈ 0.78, showing subdiffusion motions. To obtain the *D*, we fit the MSD data to MSD = 4*Dt*^0.78^ for PodJ_N droplets in the presence of SpmX(∆TM), and MSD = 4*Dt* for other samples. Only tracks with coefficient of determination (*R*^2^) ≥ 0.8 are included in the analysis.

### Partitioning analysis of molecules into the condensed phase

Partitioning analysis was used to investigate the phase distribution of molecules. The partitioning coefficient, *K*, was defined using Eq. (3):

3 $$K=\frac{{I}_{{{{{{\rm{out}}}}}}}}{{I}_{{{{{{\rm{in}}}}}}}}$$

where *I*_out_ and *I*_in_ are the average fluorescence intensity outside and inside the condensates, respectively. The fluorescence intensity data were obtained using the ROI manager tool of Fiji/ImageJ, and the same‐sized regions of at least 30 droplets and background from three independent experiments were selected in each sample. The partitioning coefficient is often expressed as Ln *K*, where a negative value indicates the proteins are likely to partitioning into the condensed phase.

### Fluorescence recovery after photobleaching (FRAP)

FRAP was used to investigate the dynamic internal rearrangement and the internal-external exchange of molecules within PodJ condensates. The in vitro FRAP analysis of liquid droplets formed by YFP-PodJ_N or its variants was performed using a Nikon A1R^+^ confocal laser scanning microscope with a 100× oil immersion objective lens. The fluorescence signal within the selected regions of protein droplets was bleached using a 488-nm laser at 50% laser power for approximately 5 s. After photobleaching, time-lapse images were captured at a rate of 1 s for approximately 5 min. The droplets with diameters ~5 µm were selected for assays at ~25 °C.

For the in vivo FRAP analysis, *C. crescentus* cells (NA1000, *pBXMCS2-P*_*xyl*_*-yfp-podJ_N* and ∆*spmX*, *pBXMCS2-P*_*xyl*_*-yfp-podJ_N*) and *E. coli* cells containing plasmid (pCDF-YFP-PodJ, pCDF-YFP-PodJ_N, pCDF-YFP-PodJ_CC1–3_, pCDF-YFP-PodJ_CC4–6_, or pCDF-YFP-PodJ_IDR_) were induced and immobilized on a 1.5% (w/v) agarose pad. Due to the relatively small sizes of *C. crescentus* cell poles (diameter: 0.2–1 µm) and the requirement for a relatively large bleach region (diameter: ≥0.4 µm), the PodJ proteins were expressed from high copy plasmids and driven by the P_*xylX*_ promoter as described above. The fluorescence signal within the selected region was bleached using a 488-nm laser at 50% laser power for approximately 2 s. After photobleaching, time-lapse images were captured every 2 s for about 5 min at 28 °C.

For each indicated time point (*t*), the fluorescence intensity within the bleached region was normalized to the fluorescence intensity of a nearby, unbleached region. The normalized fluorescence intensity of pre-bleaching region was set to 100% and the normalized fluorescence intensity at each time point (*I*_*t*_) was used to calculate the fluorescence recovery using Eq. (4):

4 $${{{{{\rm{Normalized}}}}}}\,{{{{{\rm{FRAP}}}}}}(t)=\frac{{I}_{t}}{{I}_{{{{{{\rm{pre}}}}}}-{{{{{\rm{bleaching}}}}}}}}$$

GraphPad Prism 5.0 program was used to plot and analyze the FRAP experiments.

### Characterization of subcellular accumulation of PodJ

For cell imaging studies of PodJ in *C. crescentus*, recombinant strains containing a single copy of *sfgfp-podJ* (*C. crescentus* NA1000, *xylX::P*_*xyl*_*-sfgfp-podJ*; ∆*podJ*, *xylX::P*_*xyl*_*-sfgfp-podJ*; ∆*popZ, xylX::P*_*xyl*_*-sfgfp-podJ*; ∆*tipN, xylX::P*_*xyl*_*-sfgfp-podJ*; ∆*spmX, xylX::P*_*xyl*_*-sfgfp-podJ*) or its variant (∆*podJ*, *xylX::P*_*xyl*_*-sfgfp-podJ_N*) in the chromosome were cultivated overnight at 30 °C and transferred to fresh PYE medium at a ratio of 1:10 (v/v). Cells were grown to an optical density at 600 nm of 0.7, and then induced with 0.003% (w/v) xylose for 3 h before imaging.

For cell imaging studies in *E. coli* BL21(DE3), recombinant cells containing relevant expression plasmids (pCDF-YFP-PodJ, pCDF-YFP-PodJ_N, pCDF-YFP-PodJ_CC1–3_, pCDF-YFP-PodJ_CC4–6_ or pCDF-YFP-PodJ_IDR_) were cultivated overnight at 37 °C and inoculated in fresh LB medium at a ratio of 1:100 (v/v). Cells were grown to an OD600 of 0.4 and then induced with 0.1 mM IPTG for 2 h before imaging.

The *C. crescentus* and *E. coli* cells were immobilized on a 1.5% (w/v) agarose-PYE pad and a 1.5% (w/v) agarose-LB pad, respectively. Cells were then imaged with a Nikon Eclipse Ti2-E inverted fluorescence microscope equipped with an Andor iXon Ultra DU897 EMCCD camera, using a 100× oil immersion objective lens. The specimens were illuminated with a 488-nm laser for green/yellow fluorescence. All images were acquired at 25 °C with the same laser power, exposure time, gain, and offset settings. The fluorescence intensity of the cells was quantitatively analyzed using MicrobeJ^66^.

### Inclusion body detection in *E. coli*

To rule out the possibility of dysfunctional aggregation of PodJ in cells, we co-expressed YFP-PodJ together with an inclusion body marker IbpA-mCherry^44^ in *E. coli*. The recombinant cells containing pCDF-IbpA-mCherry plasmid, or those co-transformed with pBAD-YFP-PodJ, pBAD-YFP-PodJ_CC1–3_, or pBAD-YFP-PopZ plasmids, were cultivated overnight at 37 °C and inoculated in fresh LB medium at a ratio of 1:100 (v/v). Cells were grown to an OD600 of 0.4 and then induced with 0.5 mM IPTG and 5 mM L-arabinose for 2 h before imaging. The results demonstrated that PodJ/PopZ does not co-localize with IbpA, indicating that PodJ/PopZ accumulation did not form inclusion bodies in *E. coli*, whereas PodJ_CC1–3_ did (Supplementary Fig. 5c).

To further test the possibility of aggregation of PodJ variants in cells, we co-expressed YFP-PodJ truncations together with a soluble protein marker mCherry in *E. coli*. The recombinant cells containing the pBAD-mCherry plasmid, or those co-transformed with pBAD-mCherry and pCDF-YFP-PodJ, pCDF-YFP-PodJ_N, pCDF-YFP-PodJ_CC1–3,_ pCDF-YFP-PodJ_CC4–6,_ or pCDF-YFP-PodJ_IDR_ plasmids, were induced, prepared, and imaged as described above. The results demonstrated that a high proportion of aggregates formed when expressing PodJ_CC1–3_ in *E. coli* (Supplementary Fig. 5b).

### Time-lapse imaging of PodJ during the cell cycle in *C. crescentus*

For PodJ imaging throughout the *C. crescentus* cell cycle, the *C. crescentus* wild-type strain containing a sole copy of *sfgfp-podJ* in the chromosome (NA1000 ∆*podJ*, *xylX::P*_*xyl*_*-sfgfp-podJ*) was cultivated in M2G medium and induced by adding 0.003% (w/v) xylose 1 h before cell synchronization. Swarmer cells were isolated from the culture by centrifugation (20 min at 11,000 ×*g*, 4 °C) after mixing with 1 volume of Percoll (GE Healthcare). The synchronized swarmer cells expressing sfGFP-PodJ were then immobilized on a 1.5% (w/v) agarose-PYE pad containing 20 µg ml^−1^ kanamycin and 0.003% (w/v) xylose and imaged every 2 min using a Nikon Eclipse T*i*2-E time-lapse imaging system over 1–2 cell divisions at room temperature (~4 h). The fluorescence intensity and cell length were quantitatively analyzed using MicrobeJ^66^.

### Kymograph analysis

Kymographs of fluorescence intensity were acquired using the built-in kymograph function of MicrobeJ^66^. The background signal was subtracted before the kymograph analysis, and the observation of the stalk at the cell pole of *C. crescentus* was considered as the old cell pole. A pre-division cell was selected as the starting point in Supplementary Fig. 1a. Another round of kymograph analysis was performed after the first cell division.

### Transmission electron microscopy (TEM)

For PodJ condensate visualization in living cells, the *C. crescentus* NA1000 *xylX::podJ* strain or recombinant cells of *E. coli* containing YFP-PodJ expression plasmids were induced and prepared as described above. Cells were fixed with 2.5% (w/v) glutaraldehyde in 0.1 M phosphate-buffered solution (pH 7.4, PBS) overnight at 4 °C. The cells were subsequently washed with PBS buffer and dehydrated in graded ethanol or acetone solutions. After embedding in epoxide resin, 50-nm thin frozen sections were cut using a Leica UC6 ultramicrotome and mounted on carbon-coated Formvar copper TEM grids. After staining with 2% (w/v) uranyl acetate, the samples were examined using FEI Tecnai Spirit BioTWIN electron microscopy at an operating voltage of 200 kV. Images were obtained using a Gatan 832 CCD camera.

### Estimation of polar PodJ concentration

Quantitative genome-wide protein measurements revealed that there were 1747 molecules of PodJ per *C. crescentus* cell when grown to the mid-log phase in liquid PYE medium^39^. According to the quantification of intracellular sfGFP-PodJ in Supplementary Fig. 1a, we assumed that ~60% of the total PodJ protein accumulated at the new cell pole of *C. crescentus*, which has a hemispherical shell with a radius of approximately 100 nm. The protein concentration of PodJ at the cell pole was estimated based on Eq. (5):

5 $$C=\frac{N/{N}_{{{{{{\rm{A}}}}}}}}{\frac{2}{3}\pi {{{{{{\rm{R}}}}}}}^{3}}$$

where *C* is the protein concentration of PodJ at the new cell pole, *N* is the PodJ protein molecule number at the cell pole, *N*_*A*_ is the Avogadro constant (~6.022 × 10^23^), π is the mathematical constant (~3.14159), and *R* is the radius of the hemispherical shell (~100 nm). Based on this expression, *C* was calculated to be approximately 0.79 mM, a concentration that is about 300-fold higher than the minimum concentration of PodJ LLPS in vitro.

### Screening of PodJ client proteins by co-localization experiments in *E. coli*

To screen for the client proteins of PodJ, *E. coli* BL21(DE3) was used because it does not contain any homologous polarity proteins of *C. crescentus*. In total, 23 cell cycle- or polarity-related proteins were selected from the *C. crescentus* localisome^49^ (Supplementary Table 1). The expression plasmids of the tested proteins were constructed using a fluorescent tag within the pBAD or pACYC vector, while those of fluorescent-tagged PodJ proteins were constructed based on the pCDF or pBAD vector (Supplementary Table 4).

To examine the subcellular localization of the tested protein, the expression plasmid was transformed into *E. coli* BL21(DE3) and induced by 0.1 mM IPTG for the pACYC-derived vectors, and 5 mM L-arabinose for the pBAD-derived vectors at 37 °C for 2 h. To examine the subcellular localization of the tested protein in the presence of PodJ, the expression plasmids of the tested protein and PodJ were co-transformed into *E. coli* BL21(DE3) and induced by the addition of 0.1 mM IPTG and 5 mM L-arabinose simultaneously. To further determine the interaction domain in PodJ, the tested protein was co-expressed with PodJ variants instead of full-length PodJ. Cells were prepared and imaged with a Nikon Eclipse Ti2-E inverted fluorescence microscope. Fluorescence of GFP/YFP/CFP was detected using the FITC filter (Nikon, excitation filter 475/35, dichroic mirror 499, and emission filter 530/43), and fluorescence of mCherry (mChy) was detected using the TRITC filter (Nikon, excitation filter 542/20, dichroic mirror 570, and emission filter 620/52) or the Texas Red filter (Nikon, excitation filter 555/35, dichroic mirror 585, and emission filter 630/70). The fluorescence intensity along the cell length was quantitatively analyzed using MicrobeJ^66^.

We used strict criteria to determine if a tested protein was recruited by PodJ or PodJ variants: (I) the localization pattern of the tested protein changed after co-expression with PodJ or PodJ variants; (II) the two proteins were 100% co-localized in >90% *E. coli* cells. Failure to meet either of these two criteria meant that the tested protein was not directly recruited by PodJ proteins, or the recruitment was uncertain in *E. coli*. At least 200 cells were calculated for each test set.

### Time-lapse imaging of the recruitment processes of PodJ client proteins in *E. coli*

To verify the recruitment of client proteins (PleC, CpaE, FliG) by PodJ, we examined the dynamic profiles of client subcellular localizations with PodJ induction in *E. coli*. The expression plasmids of the tested client proteins and PodJ were co-transformed in *E. coli* BL21(DE3). Cells were first induced by 5 mM L-arabinose at 37 °C for client protein expression for 2 h. Then, the client-expressing cells were immobilized on a 1.5% (w/v) agarose-LB pad containing 0.5 mM IPTG (for YFP-PodJ induction) and 5 mM L-arabinose, and imaged every 5 min using a Nikon Eclipse Ti2-E time-lapse imaging system at 37 °C (~4 h). The induction of YFP was used as a negative control.

### Assessment of the recruitment of PodJ client proteins in *C. crescentus*

To analyze the PodJ recruitment of PleC, CpaE, and FliG in *C. crescentus*, recombinant strains (NA1000, *pBVMCS6-P*_*van*_*-pleC-mCherry*; ∆*podJ, pBVMCS6-P*_*van*_*-pleC-mCherry*; ∆*podJ*, *xylX::P*_*xyl*_*-sfgfp-podJ, pBVMCS6-P*_*van*_*-pleC-mCherry* for PleC; NA1000, *pBVMCS6-P*_*van*_*-mCherry-cpaE*; ∆*podJ*, *pBVMCS6-P*_*van*_*-mCherry-cpaE*; ∆*podJ*, *xylX::P*_*xyl*_*-sfgfp-podJ*, *pBVMCS6-P*_*van*_*-mCherry-cpaE* for CpaE; NA1000, *pBVMCS6-P*_*van*_*-mCherry-fliG*; ∆*podJ, pBVMCS6-P*_*van*_*-mCherry-fliG*; ∆*podJ*, *xylX::P*_*xyl*_*-sfgfp-podJ, pBVMCS6-P*_*van*_*-mCherry-fliG* for FliG) were constructed. Overnight cultures of recombinant cells were transferred into fresh M2G medium at a ratio of 1:10 (v/v) and grown to an OD600 of 0.7. The cells were then induced with 50 µM vanillate or 0.003% (w/v) xylose plus 50 µM vanillate at 30 °C for 3 h to express the client protein alone or the co-expression with PodJ. Cells were imaged with a Nikon Eclipse Ti2-E inverted fluorescence microscope as described above. The fluorescence intensity and the localization of fluorescent focus along the cell position were quantitatively analyzed with MicrobeJ^66^.

### Screening of the negative regulator for PodJ subcellular localization in *E. coli*

To screen for the negative regulator of PodJ subcellular localization, 11 polarity proteins (Supplementary Table 2) that reside at the *C. crescentus* cell poles were selected from the *C. crescentus* localisome^49^ and their effects were examined with PodJ subcellular localization. The expression plasmids of these candidate proteins were constructed and expressed with or without fluorescent-tagged PodJ in *E. coli* BL21(DE3). Cells were prepared and imaged with a Nikon Eclipse T*i*2-E inverted fluorescence microscope as described above. The fluorescence intensity along the cell length was quantitatively analyzed with MicrobeJ^66^.

The localization pattern of PodJ was observed after co-expression with these candidate proteins. Since PodJ alone is in a bipolar pattern in *E. coli*, a negative regulator was defined as the protein that inhibits or damages the bipolar localization of PodJ in *E. coli*.

### Analysis of SpmX-PodJ interaction in *C. crescentus*

To verify the protein-protein interaction between PodJ and SpmX in *C. crescentus*, three sfGFP-PodJ expressing recombinant strains (NA1000, *xylX::P*_*xyl*_*-sfgfp-podJ*, i.e., wild-type strain; ∆*spmX, xylX::P*_*xyl*_*-sfgfp-podJ*, i.e., ∆*spmX* strain; NA1000, *xylX::P*_*xyl*_*-sfgfp-podJ*, *pBVMCS6-P*_*van*_*-mCherry-spmX*, i.e., SpmX O/E strain) were constructed. The localization of PodJ with or without SpmX expression was investigated by inducing cells with 0.003% (w/v) xylose or 0.003% (w/v) xylose plus 500 µM vanillate, and imaged as described above. The fluorescence intensity along the cell length was quantitatively analyzed with MicrobeJ^66^.

### Time-lapse imaging of the dynamic SpmX-PodJ interaction in *C. crescentus*

To better understand the SpmX regulation on PodJ in vivo, we examined the dynamic profile of PodJ condensates by inducing the cells with a single copy of *mCherry-spmX*. A sfGFP-PodJ and mCherry-SpmX co-expressing recombinant strain (NA1000, *xylX::P*_*xyl*_*-sfgfp-podJ*, *vanA::P*_*van*_*-mCherry-spmX*) was constructed. For the titration of sfGFP-PodJ with mCherry-SpmX, the cells were first induced by 0.03% (w/v) xylose at 30 °C for 3 h for PodJ expression. The cells were then immobilized on a 1.5% (w/v) agarose-PYE pad containing 0.03% (w/v) xylose and different concentrations of vanillate (0, 50, 500, or 5000 µM, for mCherry-SpmX induction), and imaged every 5 min using a Nikon Eclipse Ti2-E time-lapse imaging system at 30 °C (~4 h).

For the adverse titration of mCherry-SpmX, the cells were first induced by 50 µM vanillate at 30 °C for 3 h for mCherry-SpmX expression. The cells were then immobilized on a 1.5% (w/v) agarose-PYE pad containing 50 µM vanillate and different concentrations of xylose (0, 0.003%, 0.03%, or 0.3% (w/v), for sfGFP-PodJ induction), and imaged as described above. The cell pole fluorescence intensity was quantitatively analyzed with MicrobeJ^66^.

### Statistics and reproducibility

All experiments were repeated at least three times independently. No statistical method was used to predetermine sample size and no data were excluded from the analyses. All statistical analyses were performed using the GraphPad Prism 5.0 program. Statistically significant differences were determined using Welch’s or Student’s *t*-test, or a one-way or two-way analysis of variance with Tukey corrections as indicated. Data are presented as means ± standard error of the mean with the number of experimental replicates (*n*) provided in the figures or corresponding figure legends. *P* < 0.05 was considered significant.

### Reporting summary

Further information on research design is available in the Nature Portfolio Reporting Summary linked to this article.

## Supplementary information

Supplementary Information

Description of Additional Supplementary Files

Supplementary Video 1

Supplementary Video 2

Supplementary Video 3

Reporting Summary

## Data Availability

Data supporting the findings of this study are available within the main manuscript, the Supplementary information, and the Supplementary videos. Relevant raw microscopy images are available from the corresponding author upon reasonable request. Source Data are provided with this paper.

## Source data

Source Data
