# Supplementary material for: Phase separation modulates the assembly and dynamics of a polarity-related scaffold-signaling hub
Source: Nat Commun. 2022 Nov 23;13:7181. doi: 10.1038/s41467-022-35000-2 (PMC9684454; doi:10.1038/s41467-022-35000-2)
Supplement: Supplementary file 1 — Supplementary Information [file 41467_2022_35000_MOESM1_ESM.pdf]

## Supplementary Information

# Phase separation modulates the assembly and dynamics of a polarity related scaffold-signaling hub

Wei Tan[1,#], Sihua Cheng[1,#], Yingying Li[1,#], Xiao-Yang Li[1,2], Ning Lu[1], Jingxian Sun[1], Guiyue Tang[1], Yujiao Yang[3,4], Kezhu Cai[1,5], Xuefei Li[1], Xijun Ou[6], Xiang Gao[1], Guo-Ping Zhao[1,3,7], W. Seth Childers[8], Wei Zhao[1,*]

**Supplementary figures:**

Supplementary Figure 1. PodJ accumulation in *C. crescentus* and *E. coli*.
Supplementary Figure 2. Biophysical analyses of YFP-PodJ_N droplets in vitro.
Supplementary Figure 3. Cell compartments are formed in living cells expressing PodJ or PodJ_N.
Supplementary Figure 4. CC4-6 is responsible for the formation of PodJ condensates in *E. coli*.
Supplementary Figure 5. CC1-3 phase separates at a higher concentration in vitro and forms insoluble aggregates in *E. coli*.
Supplementary Figure 6. Live cell imaging shows that PodJ recruits the client proteins in *E. coli*.
Supplementary Figure 7. Identification of the domains for client recruitment in PodJ by heterologous co-expression experiments.
Supplementary Figure 8. SpmX regulates the dissociation of PodJ condensates in living cells.
Supplementary Figure 9. SpmX regulates the subcellular formation of PodJ condensates in *C. crescentus*.
Supplementary Figure 10. SpmX negatively regulates PodJ LLPS in vitro.
Supplementary Figure 11. The negative regulation of PodJ by SpmX for client recruitment.
Supplementary Figure 12. CC4-6 is one of the interaction domains between PodJ and SpmX.
Supplementary Figure 13. SDS-PAGE analyses of the proteins used in this study.

**Supplementary tables:**

Supplementary Table 1. Screening of PodJ client proteins in *E. coli*.

Supplementary Table 2. Screening of the regulators of PodJ subcellular localization in *E. coli*.

Supplementary Table 3. Bacterial strains used in this study.

Supplementary Table 4. Plasmids used in this study.

Supplementary Table 5. Oligonucleotides used in this study.

Supplementary Table 6. Constructions of His-tagged proteins used in this study.

**Supplementary references**

## Supplementary figures

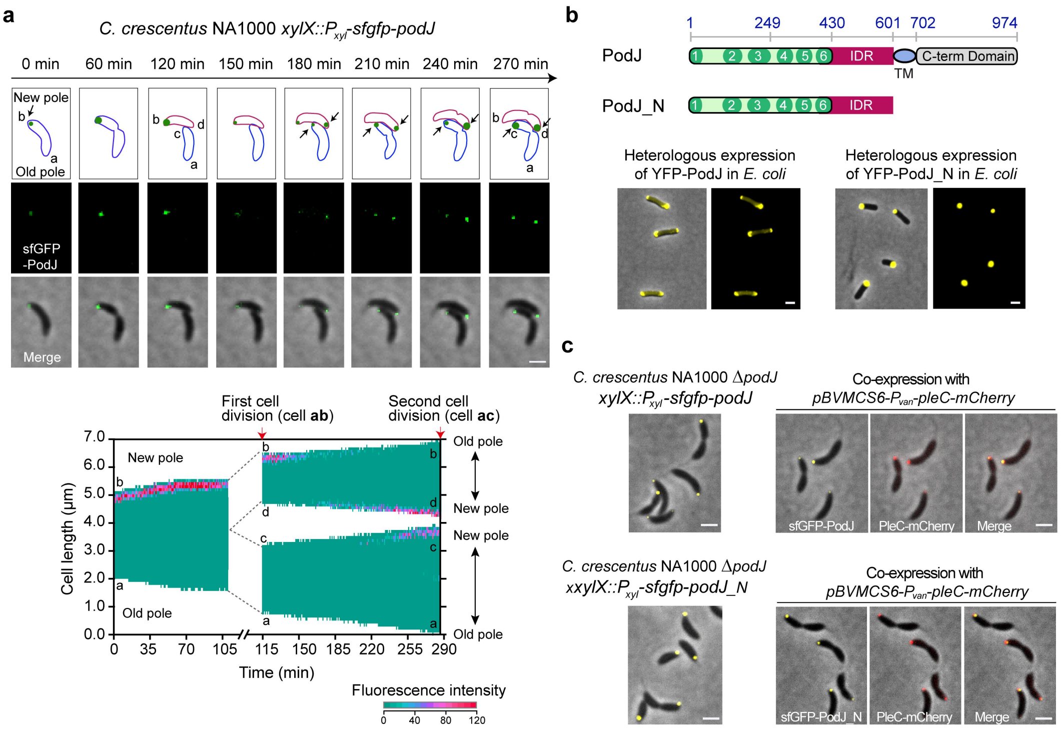


**Supplementary Figure 1**. **PodJ accumulation in *C. crescentus* and *E. coli*. a**, PodJ specifically accumulates at the new cell poles during the cell cycle. The two cell poles were distinguished by observation of the stalk at the old cell pole. A sole copy of *sfgfp-podJ* in the chromosome was induced by adding 0.003% (w/v) xylose 1 h prior to cell synchronization. Kymographs of the sfGFP-PodJ signal along the cell length over time are shown on the lower panel. Images were acquired every 2 min for *C. crescentus* cells on a PYE pad. The black arrow (upper panel), new cell pole. The red arrow (lower panel), the time point of cell division. **b**, YFP-PodJ_N (YFP-PodJ$_{1\text{-}601}$) accumulates at the cell poles in *E. coli.* Compared with the bipolar localization of YFP-PodJ, YFP-PodJ_N accumulates in a monopolar pattern in *E. coli* for unknown reasons. **c**, sfGFP-PodJ_N accumulates as the full-length YFP-PodJ in *C. crescentus.*

Fluorescence microscopy suggests that sfGFP-PodJ_N accumulates at the new cell pole and recruits a signaling protein (PleC-mCherry) as the full-length sfGFP-PodJ. PodJ and PleC were induced by 0.003% (w/v) xylose and 500 μM vanillate, respectively. All scale bars, 2 μm.

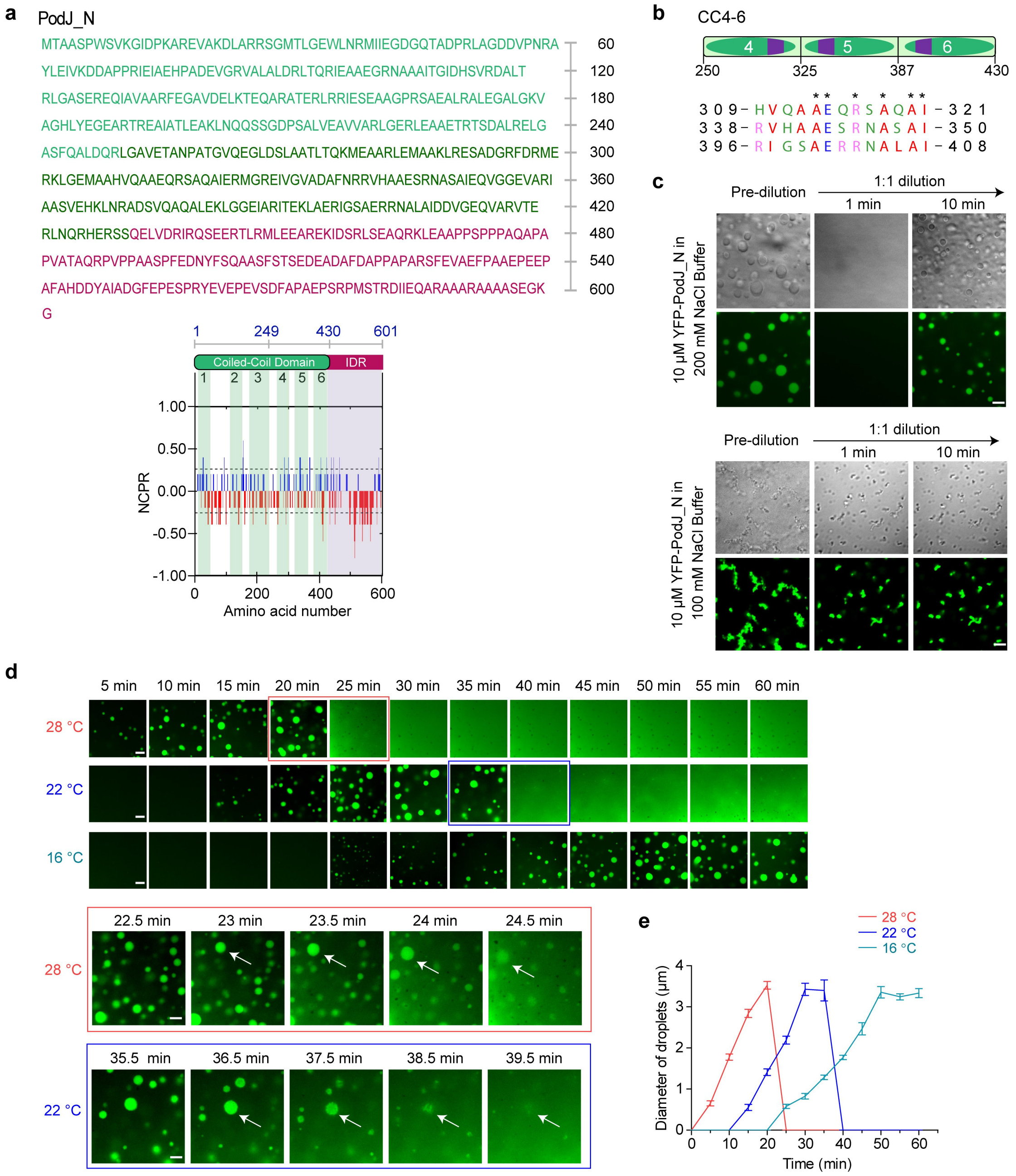


**Supplementary Figure 2**. **Biophysical analyses of YFP-PodJ_N droplets in vitro**. **a,** Amino acid sequence analyses of PodJ_N reveal that the IDR domain is rich in negatively charged residues. The PodJ domains of CC1-3, CC4-6, and IDR are colored in light green, dark green, and magenta, respectively (upper panel). CIDER[1] was used for analyzing the net charge per residue (NCPR) distribution of PodJ_N (lower panel). The NCPR plots of positively charged and negatively charged residues are indicated as blue and red, respectively. **b**, Characterization of tandem repeats in $PodJ_{CC4\text{-}6}$. Three tandem repeats (purple) characterized as AE#R#A#AI are identified

in the CC4-6 domain of PodJ. *, conserved amino acid in the tandem repeats; #, random amino acid. **c**, Reversible liquid droplets are formed by YFP-PodJ_N in a HEPES buffer (pH 7.5) containing 200 mM NaCl. YFP-PodJ_N droplets dissolved after 1:1 dilution and reassembled in 10 min in a HEPES buffer (pH 7.5) containing 200 mM NaCl. In contrast, YFP-PodJ_N in a HEPES buffer (pH 7.5) containing 100 mM NaCl formed amorphous structures which did not dissolved in the same buffer. **d**, The YFP-PodJ_N droplets emerge and disassemble faster at higher temperatures. The dynamics of YFP-PodJ_N droplets (5 μM) was monitored at different temperatures in a HEPES buffer (pH 7.5) containing 200 mM NaCl. Details of the droplet disassembly are shown with enlarged rectangles, revealing a different pattern from wetting (a phenomenon when biomolecular condensates flatten against a surface[2,3]). Arrow, the disassembling droplets. **e**, Diameter quantification of YFP-PodJ_N droplets in panel d. YFP-PodJ_N droplets (*n* = 677, 551, and 1,802 droplets, respectively; from high to low temperatures) from three independent experiments were measured. Data are means ± SEM. All scale bars, 4 μm. Source data are provided in the Source Data file.

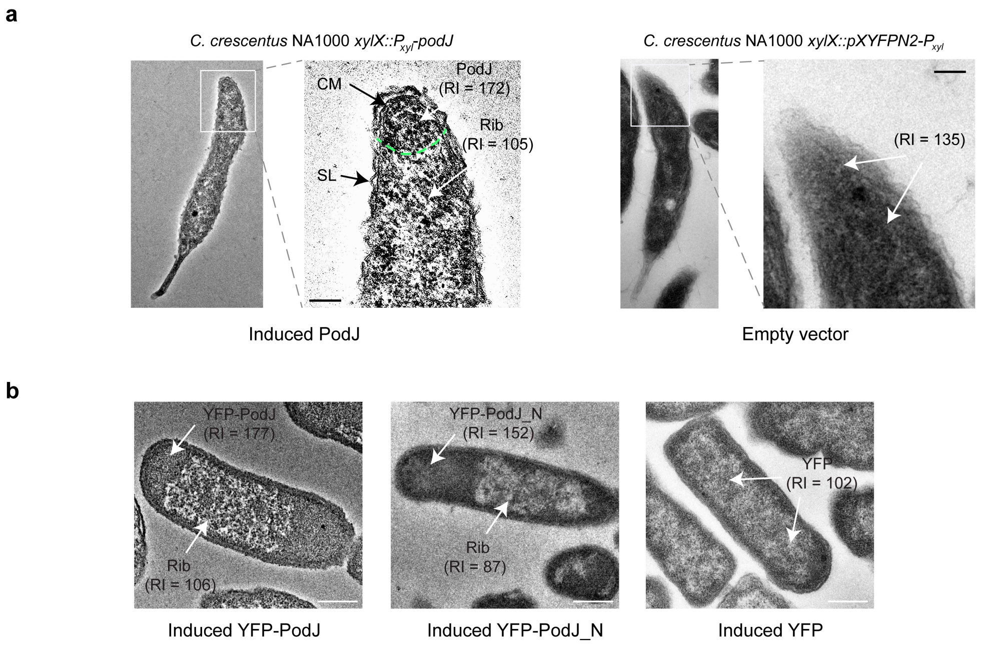


**Supplementary Figure 3. Cell compartments are formed in living cells expressing PodJ or PodJ_N. a,** TEM analysis of *C. crescentus* cells expressing PodJ (left panel) or empty vector (right panel). Both cells were induced with 0.03% (w/v) xylose for 4 hours before they were fixed. The electron density was measured as relative intensity (RI) using ImageJ. SL, surface layer; CM, cell membrane; PodJ, PodJ accumulation at the pole; Rib, ribosomes and other cell components. Scale bars, 100 nm. **b,** TEM analyses suggest that the PodJ proteins packed into the *E. coli* cell poles. *E. coli* cells expressing YFP-PodJ (left), YFP-PodJ_N (middle), and YFP (right), were induced with 0.1 mM IPTG at 37 °C for 2 h before fixation. All scale bars, 500 nm.

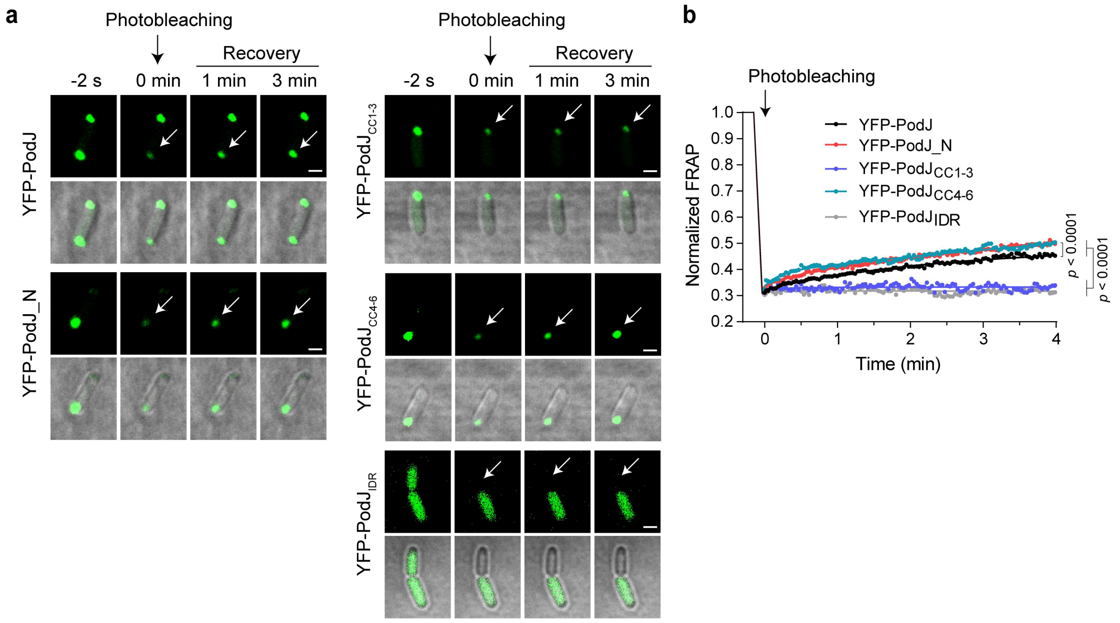


**Supplementary Figure 4. CC4-6 is responsible for the formation of PodJ condensates in *E. coli*. a**, FRAP analyses demonstrate that the fluorescence intensities of YFP-PodJ, YFP-PodJ_N, and YFP-PodJ$_{CC4-6}$ condensates were recovered after photobleaching when compared with those of YFP-PodJ$_{CC1-3}$ and YFP-PodJ$_{IDR}$ in *E. coli*. White arrow, the photobleached region. Scale bar, 1 µm. **b**, Quantitative analysis of FRAP in panel a. The FRAP recovery curves were generated by averaging the signals of YFP-PodJ or its variants ($n$ = 4 cells in YFP-PodJ$_{CC4-6}$; $n$ = 8 cells in other samples) from at least two independent experiments with nonlinear regression. The fluorescence intensity of the pre-bleached region was normalized as 100%. Data are means ± SEM and $p$ value was determined by one-way ANOVA. Source data are provided in the Source Data file.

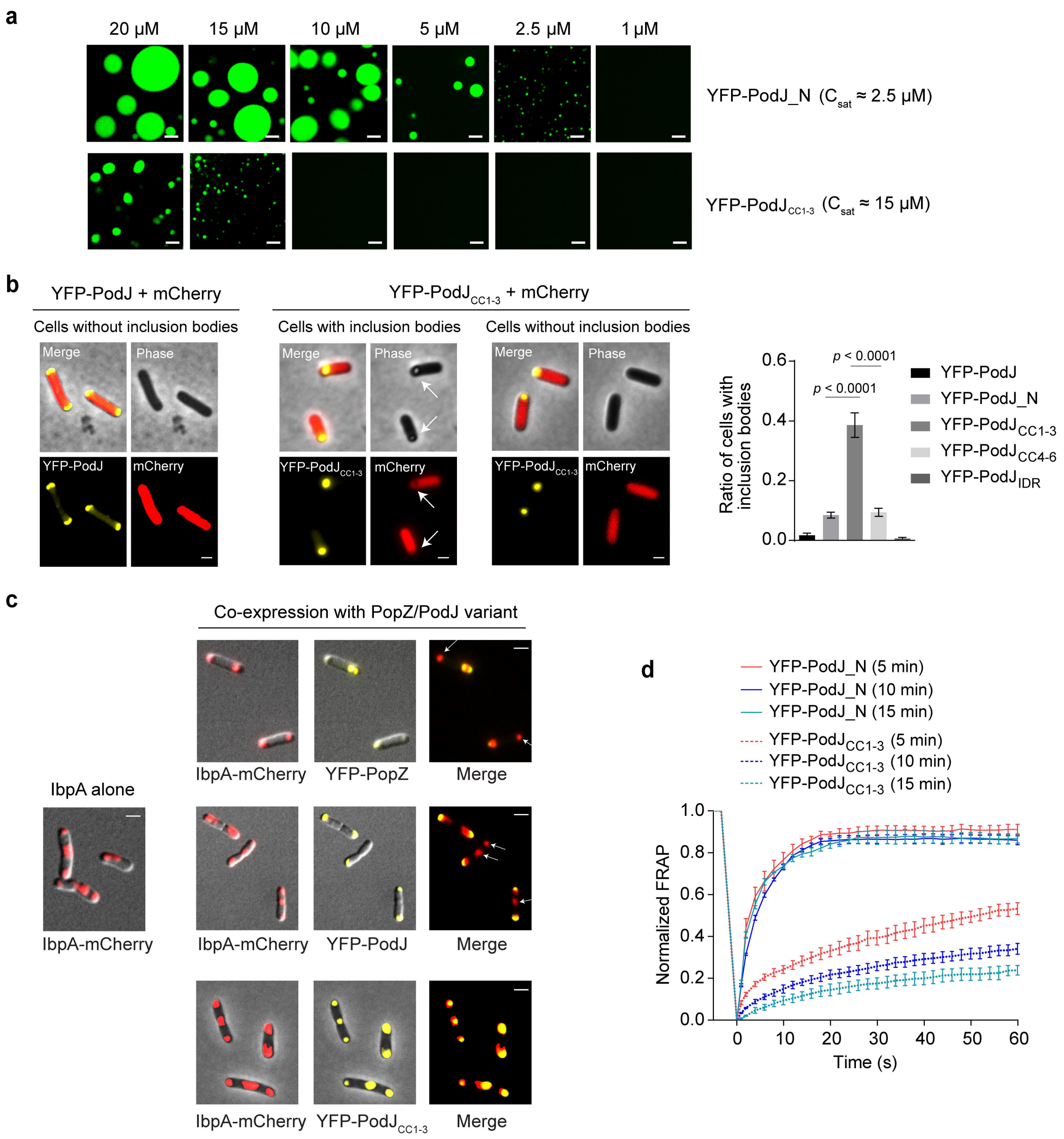


**Supplementary Figure 5. CC1-3 phase separates at a higher concentration in vitro and forms insoluble aggregates in *E. coli*. a**, YFP-PodJ$_{CC1\text{-}3}$ forms liquid droplets in vitro with a $C_{sat}$ of ~15 µM. Images were taken within 15 min after loading the ice-bathed proteins (1, 2.5, 5, 10, 15 or 20 µM) on a glass pad at 25 °C. $C_{sat}$, protein concentration of saturation for LLPS. Scale bar, 5 µm. **b**, PodJ$_{CC1\text{-}3}$ forms a large number of insoluble aggregates in *E. coli*. Co-expression assay shows that YFP-PodJ$_{CC1\text{-}3}$ tends not to let mCherry enter the polar clusters, in contrast to the full-

length YFP-PodJ. White arrows indicate the insoluble aggregates of YFP-PodJ$_{CC1\text{-}3}$. The percentages of cells with insoluble aggregates were calculated in each sample (*n* = 250, 300, 450, 350, and 250 cells; from top to bottom) from at least five independent experiments. Data are means ± SEM and *p* value was determined by one-way ANOVA. Scale bar, 1 μm. **c**, PodJ$_{CC1\text{-}3}$ co-localizes with IbpA while PodJ and PopZ do not co-localize with IbpA in *E. coli*. Co-expression of YFP-PodJ$_{CC1\text{-}3}$ and inclusion body marker protein A[4] (IbpA-mCherry) in *E. coli* showed co-localization of them, indicating that PodJ$_{CC1\text{-}3}$ forms inclusion bodies. In contrast, co-expression of YFP-PodJ/PopZ and IbpA-mCherry showed non-colocalization of these proteins, indicating that PodJ/PopZ does not form inclusion bodies. White arrows indicate the non-colocalization of proteins. Scale bar, 1 μm. **d**, PodJ$_{CC1\text{-}3}$ droplet ages over time. FRAP analyses were performed for YFP-PodJ$_{CC1\text{-}3}$ and YFP-PodJ_N droplets at different time points (5, 10 or 15 min) after loading the ice-bathed proteins (20 μM) on a glass pad at 25 °C. The recovery curves for each sample were generated by averaging the signals of droplets (*n* = 6) from three independent experiments. Data are means ± SEM. The results show that PodJ$_{CC1\text{-}3}$ droplets become less dynamic over time in contrast to PodJ_N droplets, a phenotype of protein aging that has been described previously in phase-separating proteins[5]. Source data are provided in the Source Data file.

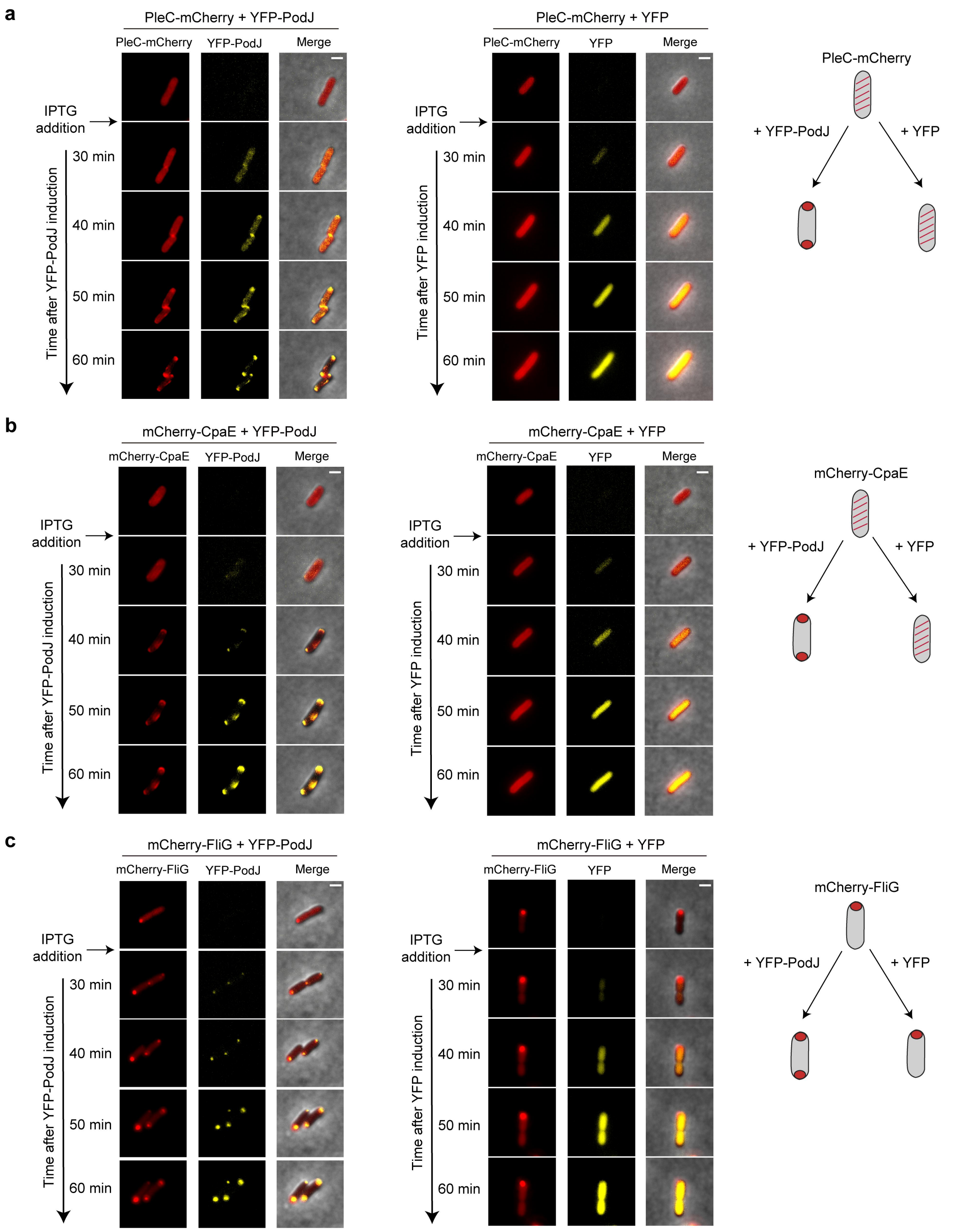


**Supplementary Figure 6. Live cell imaging shows that PodJ recruits the client proteins in *E. coli*.** The clients, including PleC (**a**), CpaE (**b**), and FliG (**c**) were pre-induced by 5 mM L-arabinose in *E. coli*, and their changes in subcellular localizations were recorded after the addition of 0.5 mM IPTG (PodJ induction). Images were

acquired every 5 min on a LB pad at 37 °C. YFP was used as the negative controls in the middle panels. Schematic diagrams are presented as the right panels illustrating the changes in subcellular localizations of the three client proteins after induction of YFP-PodJ or YFP. All scale bars, 2 μm.

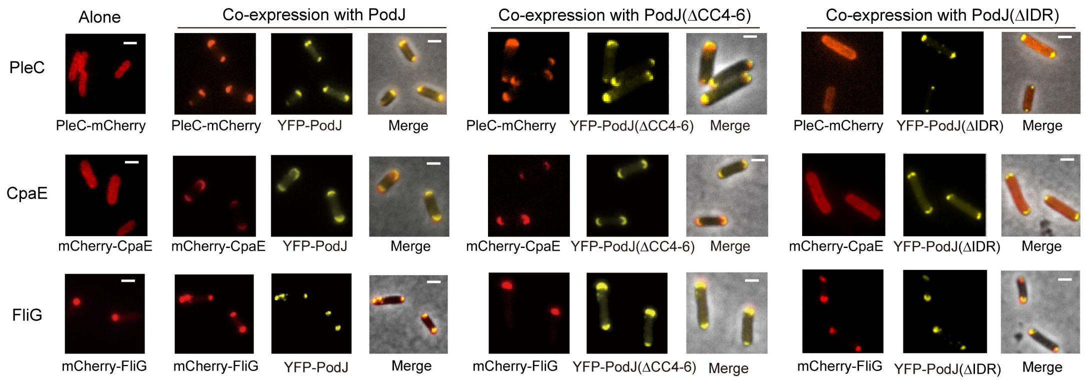


**Supplementary Figure 7. Identification of the domains for client recruitment in PodJ by heterologous co-expression experiments.** The client proteins, including PleC, CpaE, and FliG, were expressed alone or co-expressed with PodJ or PodJ variants in *E. coli* to observe their changes of subcellular localization. Quantitative results (see Fig. 4b) showed that IDR is responsible for the recruitment of PleC and CpaE, while CC4-6 is responsible for the interaction with FliG. Scale bar, 2 μm.

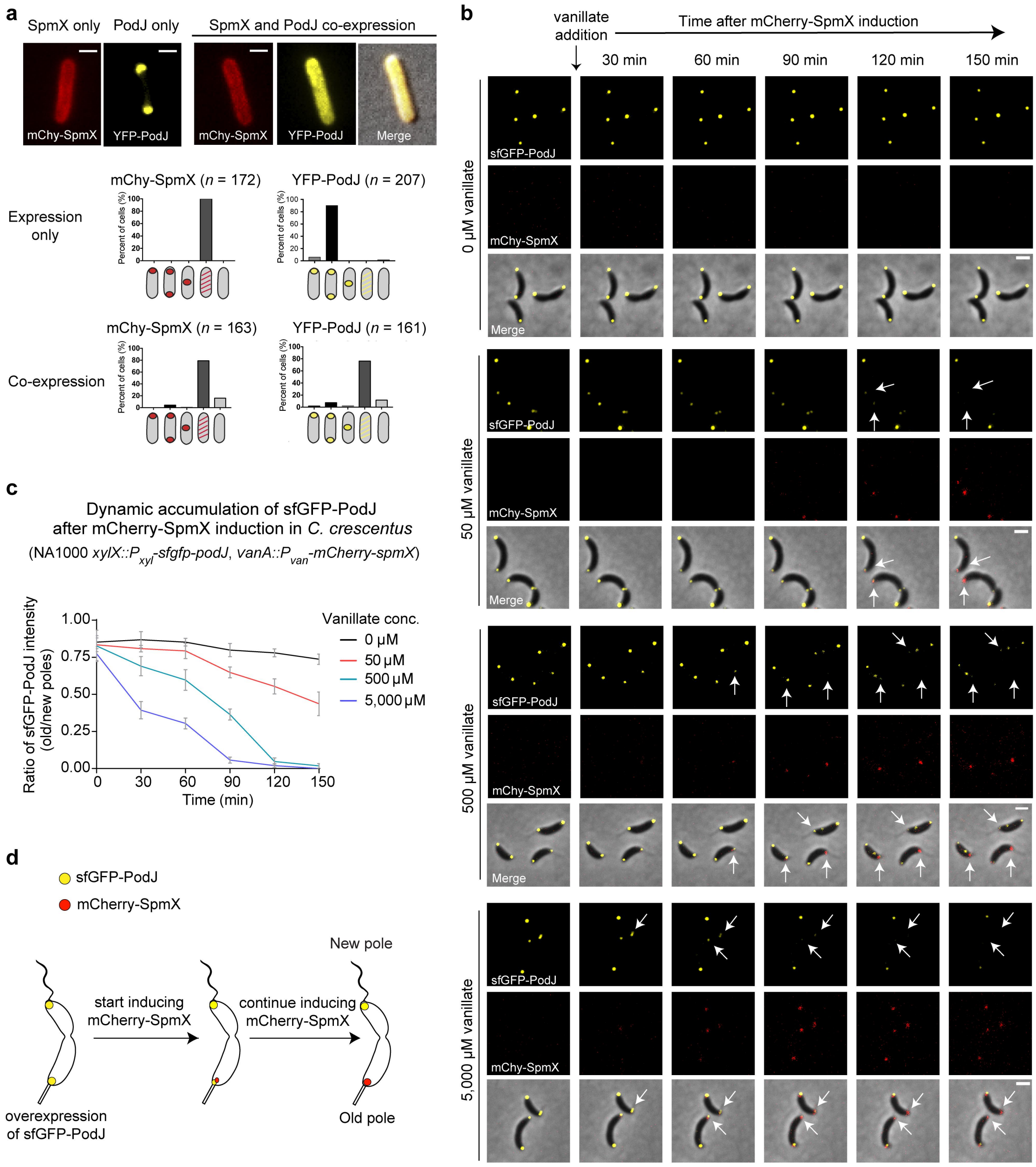


**Supplementary Figure 8. SpmX regulates the dissociation of PodJ condensates in living cells. a**, Overexpression of SpmX disrupts the polar accumulation of PodJ in *E. coli.* Co-expression of YFP-PodJ and mCherry-SpmX resulted in YFP-PodJ diffuse in *E. coli*. Histogram represents the percentage of cells with different protein localization patterns. Scale bar, 1 μm. **b**, Titration of sfGFP-PodJ with mCherry-SpmX reveals that SpmX regulates the disassembly of PodJ condensates at the cell poles of *C. crescentus*. Cells of the recombinant strain (NA1000, *xylX::P*$_{xyl}$*-sfgfp-podJ*,

*vanA::P$_{van}$-mCherry-spmX*) were first induced with 0.03% (w/v) xylose for 3 h to create the bipolar model of sfGFP-PodJ. The cells were then immobilized on a 1.5% (w/v) agarose-PYE pad containing 0.03% (w/v) xylose and different concentrations of vanillate (0, 50, 500, or 5,000 μM, for mCherry-SpmX titration). Images were acquired every 5 min at 30 °C. The fluorescence intensities of sfGFP-PodJ at both cell poles were recorded. White arrows indicate the dissociation of sfGFP-PodJ at the old cell poles. Scale bar, 2 μm. **c**, The ratio of sfGFP-PodJ intensities (old to new poles) were generated by averaging signals of sfGFP-PodJ foci (*n* = 21 cells) from three independent experiments in panel b. Data are means ± SEM. **d**, Schematic diagram illustrating the changes in subcellular localizations of sfGFP-PodJ after induction of mCherry-SpmX in *C. crescentus*. The results showed that the dissociation of sfGFP-PodJ at the cell poles (especially at the old cell pole) by mCherry-SpmX was in a concentration-dependent manner. Source data are provided in the Source Data file.

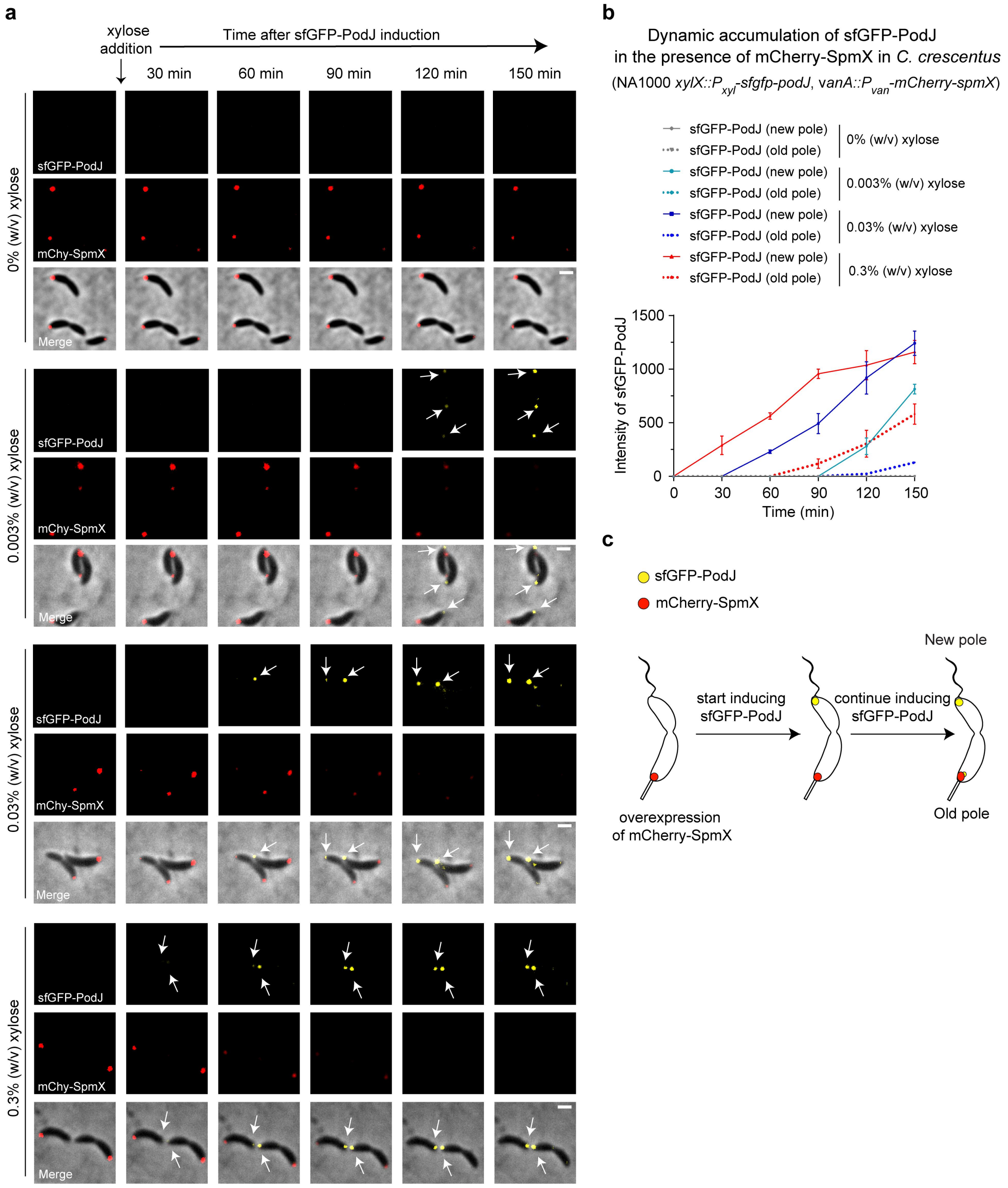


**Supplementary Figure 9. SpmX regulates the subcellular formation of PodJ condensates in *C. crescentus*. a**, Titration of mCherry-SpmX with sfGFP-PodJ reveals that SpmX regulates the formation of PodJ condensates at the cell poles of *C. crescentus*. Cells of the recombinant strain (NA1000, *xylX::P$_{xyl}$-sfgfp-podJ*, *vanA::P$_{van}$-mCherry-spmX*) were first induced with 50 µM vanillate for 3 h to create the monopolar model of mCherry-SpmX. The cells were then immobilized on a 1.5%

(w/v) agarose-PYE pad containing 50 μM vanillate and different concentrations of xylose (0, 0.003%, 0.03%, or 0.3% (w/v), for sfGFP-PodJ titration). Images were acquired every 5 min at 30 °C. The fluorescence intensities of sfGFP-PodJ at both cell poles were recorded. White arrows indicate the formation of sfGFP-PodJ condensates at the new cell poles. Scale bar, 2 μm. **b**, The sfGFP-PodJ intensities at both cell poles (old and new poles) were calculated by averaging signals of sfGFP-PodJ foci ($n$ = 21 cells) from three independent experiments in panel a. Data are means ± SEM. **c**, Schematic diagram illustrating the changes in subcellular localizations of sfGFP-PodJ in the presence of mCherry-SpmX in *C. crescentus*. The results showed that sfGFP-PodJ preferred to accumulate at the new cell pole. Source data are provided in the Source Data file.

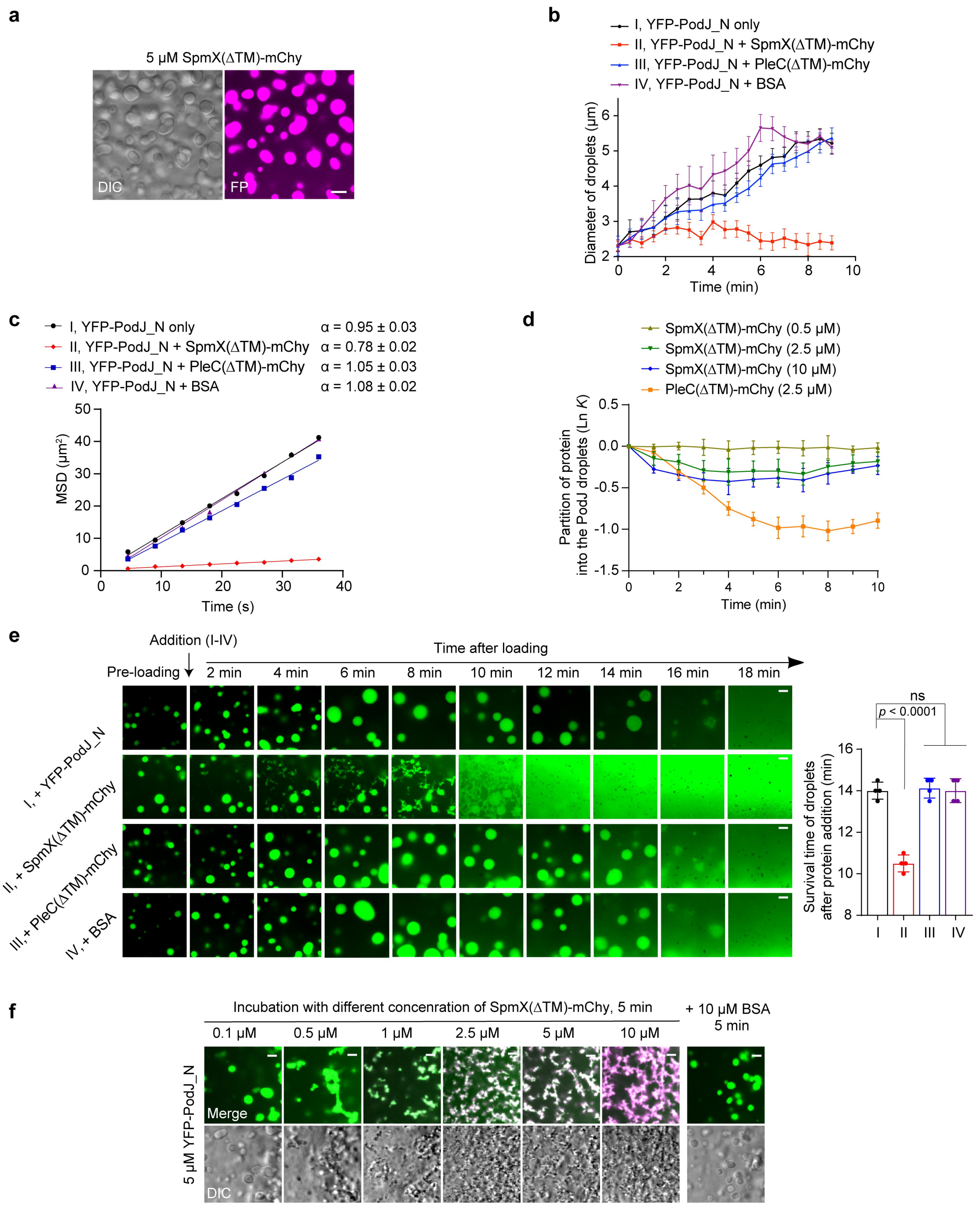


**Supplementary Figure 10. SpmX negatively regulates PodJ LLPS in vitro. a**, SpmX(ΔTM)-mCherry (5 µM) forms clear liquid droplets in vitro at 25 °C. **b**, The YFP-PodJ_N droplets were unable to grow in the presence of SpmX(ΔTM)-mCherry. Diameter analysis was performed by measurements of droplets from three independent experiments in each sample ($n$ = 1,982, 1,540, 1,112, and 786 droplets;

from top to bottom). Data are means ± SEM. **c**, The motility analyses of YFP-PodJ_N droplets by tracking single microspheres. Droplets ($n$ = 24, 25, 25, and 25 droplets; from top to bottom) from three independent experiments were analyzed, respectively. Mean-square displacement (MSD) versus time was measured for YFP-PodJ_N droplets in the presence of SpmX(ΔTM)-mCherry or PleC(ΔTM)-mCherry, using the addition of BSA as the control. The anomalous diffusion exponent (α) was obtained by fitting the dataset with log(MSD($t$)) and $t$, where the slope of the linear fit is α. The average MSD increased non-linearly (α ≈ 0.78) for YFP-PodJ_N droplets in the presence of SpmX(ΔTM)-mCherry, showing sub-diffusion behavior. Corresponding to Fig. 5f. **d**, The partitioning analysis of SpmX(ΔTM)-mCherry into YFP-PodJ_N droplets. The solution of pre-formed YFP-PodJ_N (5 μM) droplets was mixed with 0.5, 2.5, or 10 μM SpmX(ΔTM)-mCherry. The 2.5 μM of PleC(ΔTM)-mCherry was used as the positive control. Droplets ($n$ = 6) in each sample were analyzed from three independent experiments. Data are means ± SEM. The results show that SpmX did not mix with the formed PodJ droplets over time except for a very small portion of SpmX that penetrated into the droplets at the beginning, possibly before the interfacial interaction was completed. **e**, SpmX accelerates the disassembly of PodJ droplets in vitro. The solution of pre-formed YFP-PodJ_N (5 μM) droplets was mixed with 2.5 μM SpmX(ΔTM)-mCherry or PleC(ΔTM)-mCherry, using the addition of BSA as the control. Representative images are shown on the left panel. The survival time of YFP-PodJ_N droplets ($n$ = 100) in each sample were recorded and analyzed after protein

addition from four independent experiments (right panel). Data are means ± SEM and *p* value was determined by one-way ANOVA. ns, non-significant. **f**, YFP-PodJ_N results in no droplets when incubated with as low as 0.5 μM SpmX(ΔTM)-mCherry. In vitro LLPS experiments were performed by incubating YFP-PodJ_N with different concentrations of SpmX(ΔTM)-mCherry (0.1, 0.5, 1, 2.5, 5, and 10 μM). Images were acquired after 5 min of incubation. All scale bars, 5 μm. Source data are provided in the Source Data file.

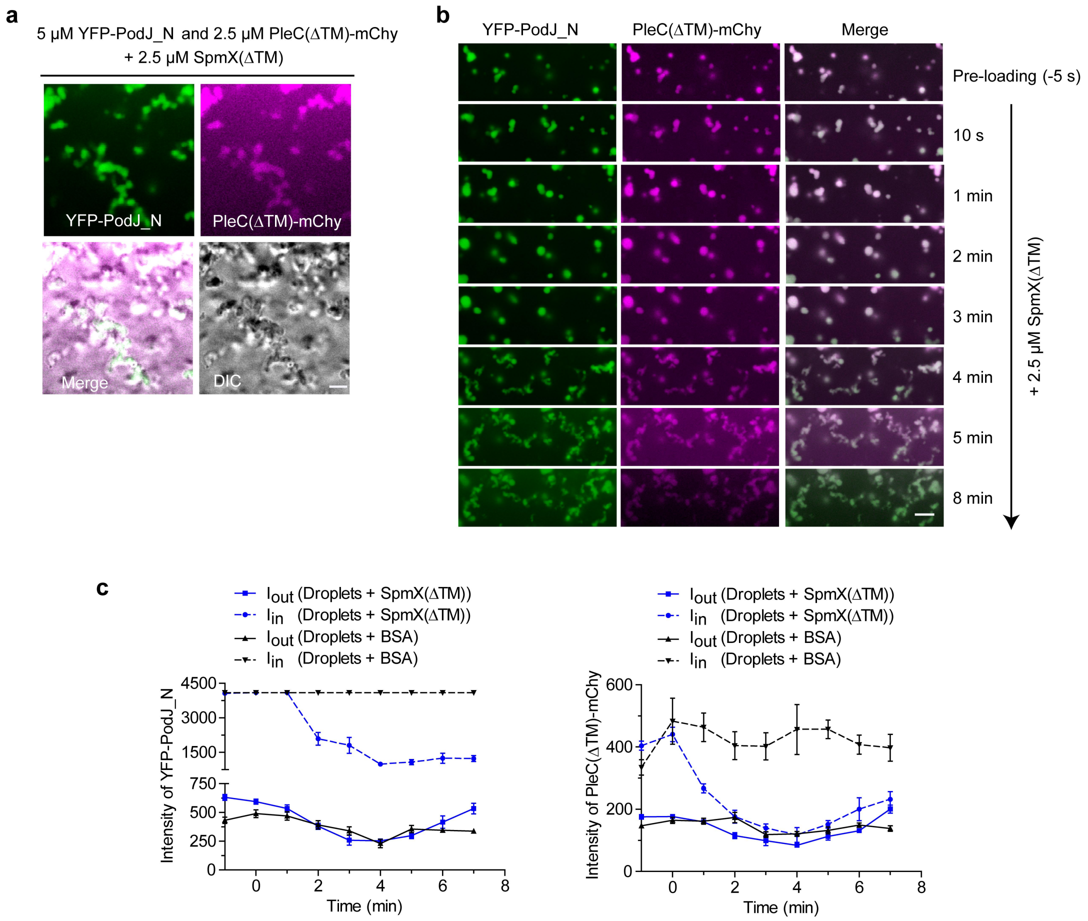


**Supplementary Figure 11. The negative regulation of PodJ by SpmX for client recruitment. a**, Incubation with SpmX(ΔTM) causes amorphous structures of YFP-PodJ_N and PleC(ΔTM)-mCherry. 5 µM YFP-PodJ_N and 2.5 µM PleC(ΔTM)-mCherry were incubated together with 2.5 µM SpmX(ΔTM)-mCherry for 15 min before imaging. **b**, The droplets of YFP-PodJ_N and PleC(ΔTM)-mCherry lose fluidity and produce amorphous structures after loading of SpmX(ΔTM). **c**, Intensity analysis of the droplets of YFP-PodJ_N and PleC(ΔTM)-mCherry with incubation of SpmX(ΔTM). Corresponding to Fig. 5j. 5 µM YFP-PodJ_N and 5 µM PleC(ΔTM)-mCherry were incubated for 15 min. Then, 2.5 µM SpmX(ΔTM) was added and images were acquired after another 5 min. The addition of 2.5 µM BSA was used as

the control. The fluorescence intensities outside ($I_{out}$) and inside ($I_{in}$) the droplets were measured using Fiji/ImageJ. Droplets ($n$ = 6) in each sample were measured from three independent experiments. Data are means ± SEM. All scale bars, 5 μm. Source data are provided in the Source Data file.

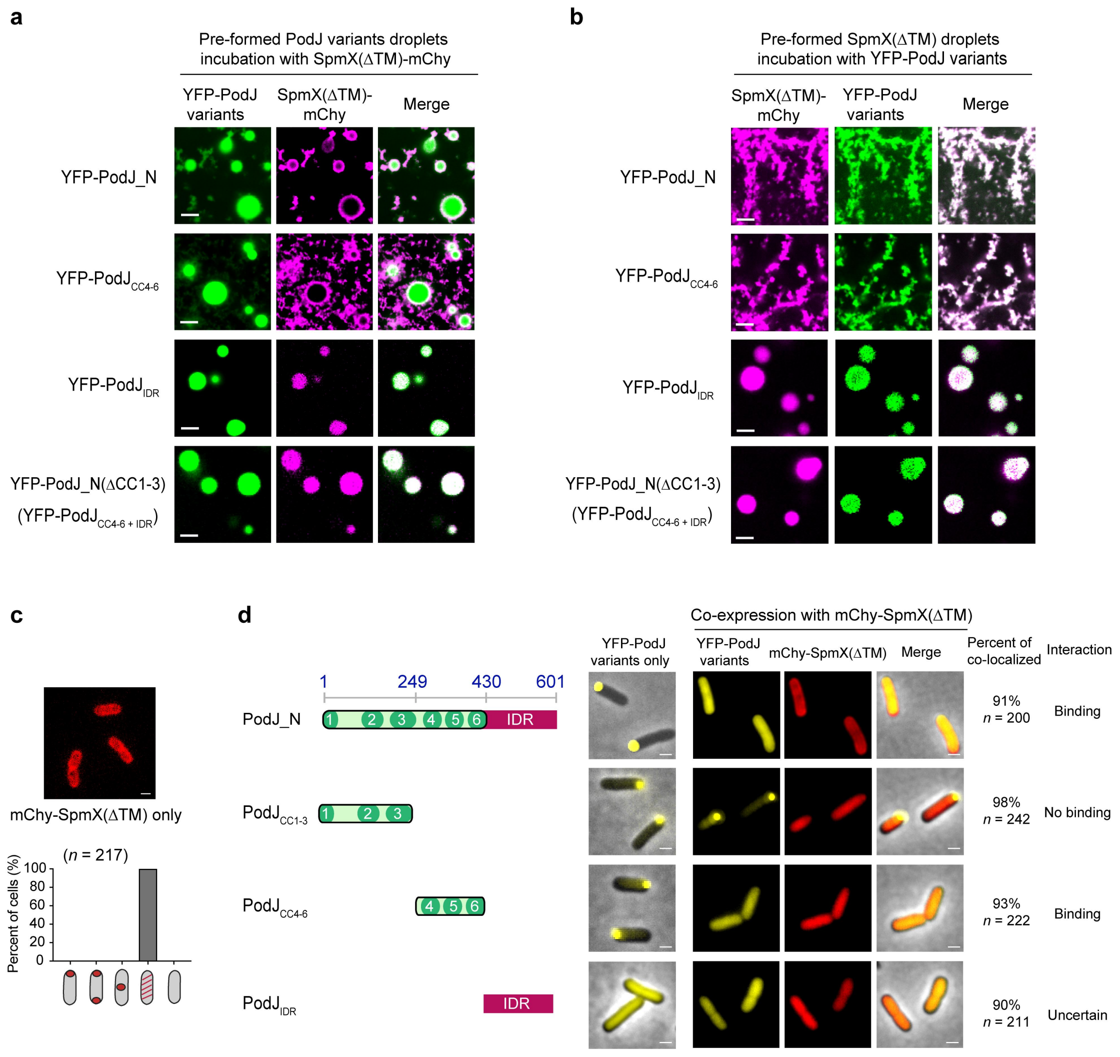


**Supplementary Figure 12. CC4-6 is one of the interaction domains between PodJ and SpmX. a**, SpmX(ΔTM) binds to the surface of PodJ$_{CC4\text{-}6}$ droplets but fuses together with PodJ$_{IDR}$. SpmX(ΔTM) interaction with PodJ$_{CC4\text{-}6+IDR}$ follows the same fusion pattern as that of SpmX(ΔTM) and PodJ$_{IDR}$. The pre-formed droplets of YFP-PodJ_N or its variants (5 μM) were incubated with 2.5 μM SpmX(ΔTM)-mCherry at 25 °C for 5 min before imaging. Scale bar, 5 μm. **b**, PodJ$_{CC4\text{-}6}$ interaction with the pre-formed SpmX(ΔTM) generates amorphous structures as that of PodJ_N. The pre-formed droplets of SpmX(ΔTM)-mCherry (5 μM) were incubated with 2.5 μM YFP-

PodJ_N and its variants at 25 °C for 5 min before imaging. Scale bar, 5 µm. **c**, mCherry-SpmX(ΔTM) expression alone is diffuse in *E. coli*. Histogram represents the percentage of cells with different protein localization patterns. Scale bar, 1 µm. **d**, Co-expression analyses of PodJ variants with SpmX(ΔTM) in *E. coli* show that CC4-6 is the interaction domain in PodJ. Representative images of cells co-expressed YFP-PodJ variants and mCherry-SpmX(ΔTM) are shown. Scale bar, 1 µm.

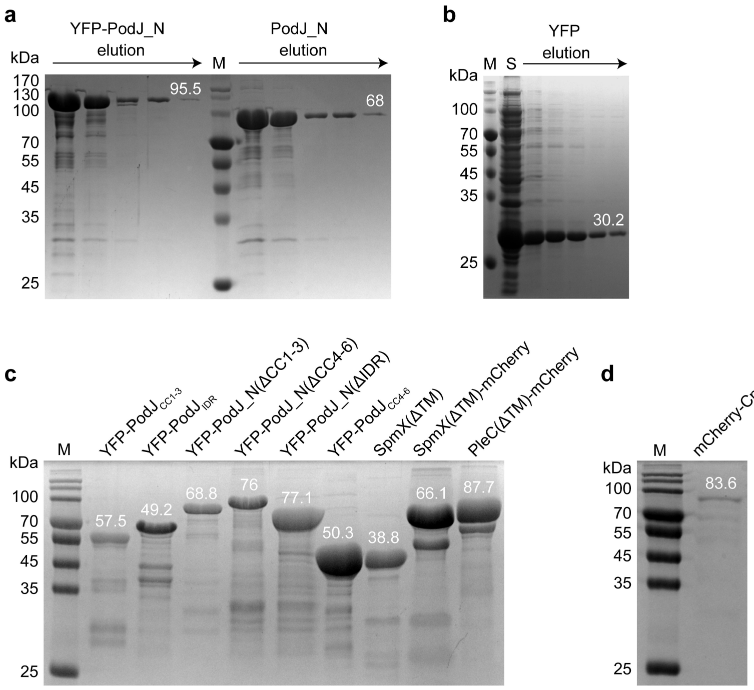


**Supplementary Figure 13. SDS-PAGE analyses of the proteins used in this study.**

The molecular masses predicted for each protein are shown on PAGE. **a,** YFP-PodJ_N and PodJ_N. **b,** YFP. **c,** YFP-PodJ variants, SpmX(ΔTM), SpmX(ΔTM)-mCherry and PleC(ΔTM)-mCherry. **d,** mCherry-CpaE. M, protein marker. S, supernatant. All the proteins were collected with purity > 80% and were stored at -80 °C before use. Concentrations of purified proteins were determined using a Bradford protein assay kit. Source data are provided in the Source Data file.

## Supplementary tables

**Supplementary Table 1. Screening of PodJ client proteins in *E. coli*.**

| Locus tag | Protein | Predicted function | Observed localization | | Co-localization relationship |
|---|---|---|---|---|---|
| | | | Expression alone | Co-expression with YFP/CFP-PodJ[a] | |
| CCNA_00268 | DnaX | DNA polymerase III subunit gamma/tau | Monopolar | Monopolar | No co-localization |
| CCNA_00437 | McpB | Methyl-accepting chemotaxis protein | Diffuse | Diffuse | No co-localization |
| CCNA_00441 | CheYI | Chemotaxis receiver domain protein | Diffuse | Diffuse | No co-localization |
| CCNA_00442 | CheAI | Chemotaxis histidine kinase protein | Diffuse | Diffuse | No co-localization |
| CCNA_00538 | | Methyl-accepting chemotaxis protein | Bipolar | Bipolar | Uncertain |
| CCNA_00947 | FlgE | Flagellar hook protein | Diffuse | Diffuse | No co-localization |
| CCNA_00950 | FliF | Flagellar M-ring protein | Diffuse | Diffuse | No co-localization |
| CCNA_00951 | FliG | Flagellar motor switch protein | Monopolar | Bipolar | Co-localized |
| CCNA_01132 | CckA | Sensory transduction histidine kinase/receiver | Diffuse | Diffuse | No co-localization |
| CCNA_01528 | FljK | Flagellin | Monopolar | Monopolar | No co-localization |
| CCNA_01918 | PopA | Cyclic di-GMP effector protein | Diffuse | Bipolar | Co-localized |
| CCNA_02546 | PleD | GGDEF/response regulator protein | Diffuse | Diffuse | No co-localization |

| | | | | | |
|---|---|---|---|---|---|
| CCNA_02547 | DivK | Response regulator receiver protein | Diffuse | Diffuse | No co-localization |
| CCNA_02567 | PleC | Sensory transduction histidine kinase | Diffuse | Bipolar | Co-localized |
| CCNA_02623 | FtsZ | Cell division protein | Patching | Patching | Uncertain |
| CCNA_02643 | FtsI | Penicillin-binding protein | Monopolar | Monopolar | No co-localization |
| CCNA_03037 | CpaF | Pilus assembly ATPase | Monopolar | Monopolar | No co-localization |
| CCNA_03038 | CpaE | Pilus assembly ATPase | Diffuse | Bipolar | Co-localized |
| CCNA_03039 | CpaD | Pilus assembly protein | Diffuse | Diffuse | No co-localization |
| CCNA_03043 | PilA | Type IV pilin protein | Diffuse | Diffuse | No co-localization |
| CCNA_03598 | DivL | Two-component sensor histidine kinase | Diffuse | Diffuse | No co-localization |
| CCNA_03868 | ParB | Chromosome partitioning protein | Diffuse | Diffuse | No co-localization |
| CCNA_03869 | ParA | Chromosome partitioning protein | Diffuse | Diffuse | No co-localization |

[a]YFP/CFP-PodJ expression alone and co-expression with potential client proteins were in a bipolar pattern in *E. coli*.

**Supplementary Table 2. Screening of the regulators of PodJ subcellular localization in *E. coli*.**

| Locus tag | Protein | Predicted function | Observed localization | | Observed PodJ localization[a] (co-expressed) |
|---|---|---|---|---|---|
| | | | Expression alone | Co-expression with YFP/CFP-PodJ[a] | |
| CCNA_00439 | McpA | Methyl-accepting chemotaxis protein | Diffuse | Diffuse | Bipolar |
| CCNA_00803 | CheW | Chemotaxis protein | Diffuse | Diffuse | Bipolar |
| CCNA_01755 | | Two component sensor histidine kinase | Bipolar | Bipolar | Bipolar |
| CCNA_01815 | NtrC | Nitrogen assimilation regulatory protein | Diffuse | Diffuse | Bipolar |
| CCNA_01817 | NtrX | Nitrogen assimilation regulatory protein | Diffuse | Diffuse | Bipolar |
| CCNA_01926 | DgcB | GGDEF diguanylate cyclase | Diffuse | Diffuse | Bipolar |
| CCNA_02255 | SpmX | Lysozyme-family localization factor | Diffuse | Diffuse | Diffuse |
| CCNA_03281 | | AsnC-family transcriptional regulator | Diffuse | Diffuse | Bipolar |
| CCNA_03333 | | PAS-family sensor histidine kinase | Diffuse | Diffuse | Bipolar |
| CCNA_03424 | TacA | AAA-family response regulator | Monopolar | Monopolar | Bipolar |
| CCNA_03584 | ChpT | Histidine phosphotransferase | Diffuse | Diffuse | Bipolar |

[a]YFP/CFP-PodJ was expressed alone in *E. coli* cells in a bipolar pattern.

**Supplementary Table 3. Bacterial strains used in this study.**

| Strains | Description[a] | Reference/Source |
|---|---|---|
| ***C. crescentus*** | | |
| NA1000 | *C. crescentus* wild-type strain | Lucy Shapiro lab |
| LS3778 | *C. crescentus* NA1000 Δ*podJ* | ref[6] |
| GB255 | *C. crescentus* NA1000 Δ*popZ* | ref[7] |
| LS4367 | *C. crescentus* NA1000 Δ*tipN* | ref[8] |
| SKR265 | *C. crescentus* NA1000 Δ*spmX* | ref[9] |
| WTS199 | $Kan^R$; *C. crescentus* NA1000 Δ*podJ*, *xylX::*$P_{xyl}$*-sfgfp-podJ* | This study |
| WTS230 | $Chl^R$; *C. crescentus* NA1000, *pBVMCS6-*$P_{van}$*-pleC-mCherry* | This study |
| WTS231 | $Chl^R$; *C. crescentus* NA1000 Δ*podJ*, *pBVMCS6-*$P_{van}$*-pleC-mCherry* | This study |
| WTS263 | $Kan^R$; *C. crescentus* NA1000, *pBXMCS2-*$P_{xyl}$*-yfp-podJ_N* | This study |
| WTS265 | $Kan^R$; *C. crescentus* NA1000 Δ*spmX*, *pBXMCS2-*$P_{xyl}$*-yfp-podJ_N* | This study |
| WTS210 | $Kan^R$; *C. crescentus* NA1000 Δ*popZ*, *xylX::*$P_{xyl}$*-sfgfp-podJ* | This study |
| WTS345 | $Kan^R$; *C. crescentus* NA1000 Δ*tipN*, *xylX::*$P_{xyl}$*-sfgfp-podJ* | This study |
| WTS211 | $Kan^R$; *C. crescentus* NA1000 Δ*spmX*, *xylX::*$P_{xyl}$*-sfgfp-podJ* | This study |
| WTS158 | $Kan^R$; *C. crescentus* NA1000, *xylX::*$P_{xyl}$*-sfgfp-podJ* | This study |
| WTS115 | $Kan^R$; *C. crescentus* NA1000, *xylX::*$P_{xyl}$*-podJ* | This study |
| WTS296 | $Kan^R$; *C. crescentus* NA1000, *xylX::pXYFPN2-*$P_{xyl}$ | This study |

| | | |
|---|---|---|
| WTS179 | $Kan^R$, $Chl^R$; *C. crescentus* NA1000, *xylX::P*$_{xyl}$*-sfgfp-podJ*, *pBVMCS6-P*$_{van}$*-mCherry-spmX* | This study |
| WTS239 | $Chl^R$; *C. crescentus* NA1000, *pBVMCS6-P*$_{van}$*-mCherry-cpaE* | This study |
| WTS240 | $Chl^R$; *C. crescentus* NA1000 Δ*podJ*, *pBVMCS6-P*$_{van}$*-mCherry-cpaE* | This study |
| WTS241 | $Kan^R$, $Chl^R$; *C. crescentus* NA1000 Δ*podJ*, *xylX::P*$_{xyl}$*-sfgfp-podJ*, *pBVMCS6-P*$_{van}$*-mCherry-cpaE* | This study |
| WTS270 | $Chl^R$; *C. crescentus* NA1000, *pBVMCS6-P*$_{van}$*-mCherry-fliG* | This study |
| WTS271 | $Chl^R$; *C. crescentus* NA1000 Δ*podJ*, *pBVMCS6-P*$_{van}$*-mCherry-fliG* | This study |
| WTS285 | $Kan^R$, $Chl^R$; *C. crescentus* NA1000 Δ*podJ*, *xylX::P*$_{xyl}$*-sfgfp-podJ*, *pBVMCS6-P*$_{van}$*-mCherry-fliG* | This study |
| WTS202 | $Kan^R$; *C. crescentus* NA1000 Δ*podJ*, *xylX::P*$_{xyl}$*-sfgfp-podJ_N* | This study |
| WTS232 | $Kan^R$, $Chl^R$; *C. crescentus* NA1000 Δ*podJ*, *xylX::P*$_{xyl}$*-sfgfp-podJ*, *pBVMCS-6-P*$_{van}$*-pleC-mCherry* | This study |
| WTS275 | $Kan^R$, $Chl^R$; *C. crescentus* NA1000 Δ*podJ*, *xylX::P*$_{xyl}$*-sfgfp-podJ_N*, *pBVMCS6-P*$_{van}$*-pleC-mCherry* | This study |
| WTS342 | $Kan^R$, $Chl^R$; *C. crescentus* NA1000 *xylX::P*$_{xyl}$*-sfgfp-podJ*, *vanA::P*$_{van}$*-mCherry-spmX* | This study |
| ***E. coli*** | | |
| DH5α | Bacterial cloning strain | Novagen |
| BL21(DE3) | Bacterial expression strain | Novagen |

[a]Abbreviations: Kan, kanamycin; Chl, Chloramphenicol; R, resistance.

**Supplementary Table 4. Plasmids used in this study.**

| Plasmid | Plasmid information[a] | Reference/Source |
|---|---|---|
| pWT188 | $Kan^R$; pBXMCS2-$P_{xyl}$-mNG-PodJ | This study |
| pWT022 | $Kan^R$; pXYFPN2-$P_{xyl}$-sfGFP-PodJ | This study |
| pWT194 | $Chl^R$; pBVMCS6-$P_{van}$-PleC-mCherry | This study |
| pWT191 | $Chl^R$; pBVMCS6-$P_{van}$-mCherry-SpmX | This study |
| pWT205 | $Kan^R$; pBXMCS2-$P_{xyl}$-YFP-PodJ | This study |
| pWT206 | $Kan^R$; pBXMCS2-$P_{xyl}$-YFP-PodJ_N | This study |
| pWT195 | $Chl^R$; pBVMCS6-$P_{van}$-mCherry-CpaE | This study |
| pWT200 | $Chl^R$; pBVMCS6-$P_{van}$-mCherry-FliG | This study |
| pWT147 | $Kan^R$; pXYFPN2-$P_{xyl}$-sfGFP-PodJ_N | This study |
| pWT243 | $Chl^R$; pVCHYN6-$P_{van}$-mCherry-SpmX | This study |
| pWT046 | $Kan^R$; pXYFPN2-$P_{xyl}$-PodJ | This study |
| pWT212 | $Kan^R$; pXYFPN2-$P_{xyl}$ | This study |
| pWT011 | $Spec^R$; pCDF-YFP-PodJ | This study |
| pWT038 | $Amp^R$; pBAD-CFP-PodJ | This study |
| pWT009 | $Chl^R$; pACYC-mCherry-CpaE | This study |
| pWT244 | $Spec^R$; pCDF-IbpA-mCherry | This study |
| pWT245 | $Amp^R$; pBAD-YFP-PodJ | This study |

| | | |
|---|---|---|
| pWT246 | $Amp^R$; pBAD-YFP-PodJ$_{CC1-3}$ | This study |
| pWT015 | $Chl^R$; pACYC-FtsZ-mCherry | This study |
| pWT247 | $Amp^R$; pBAD-CckA-mCherry | This study |
| pWT248 | $Amp^R$; pBAD-DivK-mCherry | This study |
| pWT249 | $Amp^R$; pBAD-DivL-mCherry | This study |
| pWT250 | $Chl^R$; pACYC-PleD-mCherry | This study |
| pWT014 | $Chl^R$; pACYC-PopA-mCherry | This study |
| pWT080 | $Amp^R$; pBAD-PleC-mCherry | This study |
| pWT081 | $Amp^R$; pBAD-mCherry-PopZ | This study |
| pWT251 | $Spec^R$; pCDF-mCherry-PopZ | This study |
| pWT013 | $Chl^R$; pACYC-mCherry-SpmX | This study |
| pWT012 | $Amp^R$; pBAD-SpmX(ΔTM)-mCherry | This study |
| pWT252 | $Chl^R$; pACYC-CFP-ParB | This study |
| pWT253 | $Chl^R$; pACYC-ParA-mCherry | This study |
| pWT207 | $Amp^R$; pBAD-YFP-PopZ | This study |
| pWT003 | $Spec^R$; pCDF-YFP-PodJ$_{CC1-3}$ | This study |
| pWT082 | $Spec^R$; pCDF-YFP | This study |
| pWT174 | $Spec^R$; pCDF-YFP-PodJ$_{CC4-6}$ | This study |
| pWT173 | $Spec^R$; pCDF-YFP-PodJ$_{IDR}$ | This study |
| pWT079 | $Spec^R$; pCDF-YFP-PodJ_N | This study |

| | | |
|---|---|---|
| pWT165 | $Spec^R$; pCDF-YFP-PodJ(ΔIDR) | This study |
| pWT024 | $Spec^R$; pCDF-YFP-PodJ(ΔCC4-6) | This study |
| pWT004 | $Amp^R$; pBAD-mCherry-$SpmX_{1-356}$ | This study |
| pWT005 | $Amp^R$; pBAD-PopA-mCherry | This study |
| pWT006 | $Amp^R$; pBAD-FtsZ-mCherry | This study |
| pWT007 | $Amp^R$; pBAD-mCherry-CpaE | This study |
| pWT027 | $Amp^R$; pBAD-FljK-mCherry | This study |
| pWT028 | $Amp^R$; pBAD-FlgE-mCherry | This study |
| pWT029 | $Amp^R$; pBAD-CpaF-mCherry | This study |
| pWT030 | $Amp^R$; pBAD-PilA-mCherry | This study |
| pWT031 | $Amp^R$; pBAD-McpA-mCherry | This study |
| pWT032 | $Amp^R$; pBAD-McpB-mCherry | This study |
| pWT033 | $Amp^R$; pBAD-CheAI-mCherry | This study |
| pWT034 | $Amp^R$; pBAD-CheYI-mCherry | This study |
| pWT057 | $Amp^R$; pBAD-mCherry-FliF | This study |
| pWT059 | $Amp^R$; pBAD-mCherry-CCNA_03333 | This study |
| pWT060 | $Amp^R$; pBAD-mCherry-ChpT | This study |
| pWT061 | $Amp^R$; pBAD-mCherry-FtsI | This study |
| pWT062 | $Amp^R$; pBAD-mCherry-CCNA_00538 | This study |
| pWT063 | $Amp^R$; pBAD-CheW-mCherry | This study |

| | | |
|---|---|---|
| pWT064 | $Amp^R$; pBAD-FliF-mCherry | This study |
| pWT025 | $Amp^R$; pBAD-mCherry-FliG | This study |
| pWT066 | $Amp^R$; pBAD-CCNA_01755-mCherry | This study |
| pWT067 | $Amp^R$; pBAD-NtrC-mCherry | This study |
| pWT068 | $Amp^R$; pBAD-NtrX-mCherry | This study |
| pWT069 | $Amp^R$; pBAD-DgcB-mCherry | This study |
| pWT070 | $Amp^R$; pBAD-CpaD-mCherry | This study |
| pWT071 | $Amp^R$; pBAD-CCNA_03281-mCherry | This study |
| pWT072 | $Amp^R$; pBAD-TacA-mCherry | This study |
| pWT073 | $Amp^R$; pBAD-DnaX-mCherry | This study |
| pWT076 | $Amp^R$; pBAD-CCNA_00538-mCherry | This study |
| pWT240 | $Amp^R$; pBAD-mCherry | This study |
| pWT001 | $Amp^R$; pTEV5-PodJ_N | This study |
| pWT086 | $Amp^R$; pTEV5-YFP-PodJ_N(ΔCC1-3) | This study |
| pWT037 | $Amp^R$; pTEV5-YFP-PodJ_N | This study |
| pWT039 | $Amp^R$; pTEV5-YFP | This study |
| pWT170 | $Amp^R$; pTEV5-YFP-PodJ_N(ΔIDR) | This study |
| pWT141 | $Amp^R$; pTEV5-YFP-$PodJ_{CC1-3}$ | This study |
| pWT142 | $Amp^R$; pTEV5-YFP-$PodJ_{CC4-6}$ | This study |
| pWT144 | $Amp^R$; pTEV5-YFP-$PodJ_{IDR}$ | This study |

| | | |
|---|---|---|
| pWT146 | $Amp^R$; pTEV5-YFP-PodJ_N(ΔCC4-6) | This study |
| pET28a(+) | $Kan^R$; bacterial protein expression vector | Novagen |
| pWT150 | $Kan^R$; pET28a-mCherry-SpmX(ΔTM) | This study |
| pWT154 | $Kan^R$; pET28a-mCherry-CpaE | This study |
| pWT156 | $Kan^R$; pET28a-PleC-mCherry | This study |
| pWT169 | $Kan^R$; pET28a-PleC(ΔTM)-mCherry | This study |
| pWT187 | $Kan^R$; pET28a-mCherry-PopZ | This study |
| pWT189 | $Kan^R$; pET28a-SpmX(ΔTM) | This study |
| pWT190 | $Kan^R$; pET28a-SpmX(ΔTM)-mCherry | This study |
| pWT209 | $Kan^R$; pET28a-mCherry | This study |

[a]Abbreviations: Kan, kanamycin; Chl, chloramphenicol; Spec, spectinomycin; Amp, ampicillin; R, resistance.

**Supplementary Table 5. Oligonucleotides used in this study.**

| Primer | Sequence | Constructs |
|---|---|---|
| WTP139 | gagacgaccatatgacggcggcttcgccatg | pWT046 |
| WTP140 | gccgccgtcatatggtcgtctccccaaaactc | |
| WTP602 | gagacgaccatgccttaattaatatgcatgg | pWT212 |
| WTP603 | ttaaggcatggtcgtctccccaaaactcgagc | |
| WTP464 | aggcaagggctaagccttaattaatatgca | pWT147 |
| WTP465 | ttaaggcttagcccttgccttccgaggcgg | |
| WTP528 | gaactgtacaaataagccttaattaatatgca | pWT166 |
| WTP529 | ttaaggcttatttgtacagttcatccatac | |
| WTP548 | ggatcagcgccaccggtcggccaccatgcgtaaaggcgaagagct | pWT180 |
| WTP549 | taattaaggcttatttgtacagttcatccatacca | |
| WTP550 | gaactgtacaaataagccttaattaatatgcatggtaccttaagatct | |
| WTP551 | ctttacgcatggtggccgaccggtggcgctgatccagggcctgga | |
| WTP007 | ggatcagcgctaagcagatctcaattggat | pWT003 |
| WTP008 | gatctgcttagcgctgatccagggcctggaac | |
| WTP459 | ggatcagcgccaggaactggtcgaccgcat | pWT024 |
| WTP460 | ccagttcctggcgctgatccagggcctggaac | |
| WTP137 | aggagatataatgacggcggcttcgccatg | pWT045 |

| | | |
|---|---|---|
| WTP138 | gccgccgtcattatatctccttcttatactt | |
| WTP147 | aggcaagggctaattaacctaggctgctgc | pWT079 or pWT049 |
| WTP148 | aggttaattagcccttgccttccgaggcgg | |
| WTP254 | gctgtacaagtaagcagatctcaattggat | pWT082 |
| WTP255 | gatctgcttacttgtacagctcgtccatgc | |
| WTP526 | gaacgttccagctaagcagatctcaattggat | pWT164 |
| WTP527 | gatctgcttagctggaacgttcgtggcgtt | |
| WTP524 | gaacgttccagcggcaaggccaagtcggcgaa | pWT165 |
| WTP525 | tggccttgccgctggaacgttcgtggcgtt | |
| WTP530 | aggcaagggctaagcagatctcaattggata | pWT167 |
| WTP531 | gatctgcttagcccttgccttccgaggcggc | |
| WTP009 | ggtggagccgtaagaagcttggctgttttggcggatgagagaagat | pWT004 |
| WTP010 | tgctcaccatttaattcctcctgttagcccaaaaaacgggtatggaga | |
| WTP011 | gaggaattaaatggtgagcaagggcgaggag | |
| WTP012 | aagcttcttacggctccaccagcggcac | |
| WTP013 | cgctgaagcgcgaggcgggccaccggtcggccacc | pWT005 |
| WTP014 | attcgggcgtcaaccgccatttaattcctcctgttagcccaaaaaacgggtatgg | |
| WTP015 | gggctaacaggaggaattaaatggcggttgacgcc | |
| WTP016 | accatggtggccgaccggtggcccgcctcgcg | |

| | | |
|---|---|---|
| WTP017 | tcctgcgccgcctggccaaccaccggtcggccacc | pWT006 |
| WTP018 | gcggaaagagaaatagccatttaattcctcctgttagcccaaaaaacgg | |
| WTP019 | gggctaacaggaggaattaaatggctatttctctttccgcg | |
| WTP020 | accatggtggccgaccggtggttggccaggcg | |
| WTP021 | gaagaagtaggaagcttggctgttttggcggatgagag | pWT007 |
| WTP022 | tgctcaccatttaattcctcctgttagcccaaaaaacgggtatggaga | |
| WTP023 | gaggaattaaatggtgagcaagggcgaggag | |
| WTP024 | gccaagcttcctacttcttcttgaacaggcccgagaaca | |
| WTP064 | cctgttccgtcaccggtcggccaccatgg | pWT027 |
| WTP065 | tcagcgccatttaattcctcctgttagcccaaaaaacgggtatggagaaacagtagagag | |
| WTP066 | gaggaattaaatggcgctgaacagcatcaatacgaacg | |
| WTP067 | ccgaccggtgacggaacaggctcaggatcgagg | |
| WTP068 | tattaagcgccaccggtcggccaccatgg | pWT028 |
| WTP069 | tgatgctcatttaattcctcctgttagcccaaaaaacgggtatggag | |
| WTP070 | gaggaattaaatgagcatcaacagcgccatgct | |
| WTP071 | ccgaccggtggcgcttaatattcaagagttcctcaagcatctggt | |
| WTP072 | cgcggcggagcaccggtcggccaccatgg | |
| WTP073 | ttccgaacatttaattcctcctgttagcccaaaaaacgggtatggag | |
| WTP074 | gaggaattaaatgttcggaaagcgcgactcgt | |

| | | |
|---|---|---|
| WTP075 | ccgaccggtgctccgccgcgtcgaggg | pWT029 |
| WTP076 | ggctggcacccaccggtcggccaccatgg | pWT030 |
| WTP077 | acttggtcatttaattcctcctgttagcccaaaaaacgggtatggag | |
| WTP078 | gaggaattaaatgaccaagttcgtcacgcgct | |
| WTP079 | ccgaccggtgggtgccagccgccgt | |
| WTP080 | ggaggaattccaccggtcggccaccatggtgag | pWT031 |
| WTP081 | tcgccaacatttaattcctcctgttagcccaaaaaacgggtatggag | |
| WTP082 | gaggaattaaatgttggcgatccgtgggcac | |
| WTP083 | ccgaccggtggaattcctcccaaccatccgaggcg | |
| WTP084 | ggaagaattccaccggtcggccaccatgg | pWT032 |
| WTP085 | cggtccccatttaattcctcctgttagcccaaaaaacgggtatggag | |
| WTP086 | gaggaattaaatggggaccgccatgaaccag | |
| WTP087 | ccgaccggtggaattcttcccactcttccttgaccgcgaccg | |
| WTP088 | cgcagcggagcaccggtcggccaccatgg | pWT033 |
| WTP089 | gctcgtccatttaattcctcctgttagcccaaaaaacgggtatggagaaacagtagagag | |
| WTP090 | gaggaattaaatggacgagctagaggccatcaaggtcaccttct | |
| WTP091 | ccgaccggtgctccgctgcgatcaggctgg | |
| WTP092 | cgtcgccgcccaccggtcggccaccatgg | pWT034 |
| WTP093 | tacgcgtcacttaattcctcctgttagcccaaaaaacgggtatggaga | |

| | | |
|---|---|---|
| WTP094 | gaggaattaagtgacgcgtacggttctcacggt | |
| WTP095 | ccgaccggtgggcggcgacgcggcgga | |
| WTP164 | cgagtcgacctaagaagcttggctgttttggcggatgagag | pWT057 |
| WTP165 | agctttccacggtggccgaccggtgcttg | |
| WTP166 | gtcggccaccgtggaaagctttctgggttcaatcagg | |
| WTP167 | aagcttcttaggtcgactcgtgcagccagt | |
| WTP172 | ggaggcgtaataagaagcttggctgttttggcggatgagag | pWT059 |
| WTP173 | tcgtactcaaggtggccgaccggtgct | |
| WTP174 | gtcggccaccttgagtacgagaccctgtccggcgagg | |
| WTP175 | aagcttcttattacgcctcccccagcatccg | |
| WTP176 | cccggcgtaataagaagcttggctgttttggcggatgagagaagattttcag | pWT060 |
| WTP177 | tctcggtcatggtggccgaccggtgct | |
| WTP178 | gtcggccaccatgaccgagaccgtcaccgagac | |
| WTP179 | aagcttcttattacgccgggacccaggc | |
| WTP180 | gggcctatgataagaagcttggctgttttggcggatgagag | pWT061 |
| WTP181 | agaggctcatggtggccgaccggtgct | |
| WTP182 | gtcggccaccatgagcctctcgaacctgggtcc | |
| WTP183 | aagcttcttatcataggcccgcctccggc | |
| WTP184 | ggcggcctagtaagaagcttggctgttttggcggatgagaga | pWT062 |

| | | |
|---|---|---|
| WTP185 | aaatgcgcaaggtggccgaccggtgct | |
| WTP186 | gtcggccaccttgcgcatttcgggaaccttgaacctct | |
| WTP187 | aagcttcttactaggccgccttctggcgca | |
| WTP188 | cgaagcggcccaccggtcggccaccatgg | pWT063 |
| WTP189 | cggtcatcacttaattcctcctgttagcccaaaaaacgggtatggaga | |
| WTP190 | gaggaattaagtgatgaccgacaacaccgcgc | |
| WTP191 | ccgaccggtgggccgcttcgctgacctgg | |
| WTP192 | cgagtcgacccaccggtcggccaccatgg | pWT064 |
| WTP193 | agctttccacttaattcctcctgttagcccaaaaaacgggtatggaga | |
| WTP194 | gaggaattaagtggaaagctttctgggttcaatcaggcagt | |
| WTP195 | ccgaccggtgggtcgactcgtgcagccagttacg | |
| WTP200 | cgccgcatcccaccggtcggccaccatgg | pWT066 |
| WTP201 | tggtggtcacttaattcctcctgttagcccaaaaaacgggtatggagaaacagtagag | |
| WTP202 | gaggaattaagtgaccaccaacagcgcgacaag | |
| WTP203 | ccgaccggtgggatgcggcgcgcgg | |
| WTP204 | cggtcgtcgccaccggtcggccaccatgg | pWT067 |
| WTP205 | cggcgttcatttaattcctcctgttagcccaaaaaacgggtatggag | |
| WTP206 | gaggaattaaatgaacgccgcgagcaagaaaatcc | |
| WTP207 | ccgaccggtggcgacgaccgcgggtca | |

| | | |
|---|---|---|
| WTP208 | tgaggaagagcaccggtcggccaccatggtgagcaagggc | pWT068 |
| WTP209 | cggcgctcatttaattcctcctgttagcccaaaaaacgggtatggag | |
| WTP210 | gaggaattaaatgagcgccgacgttcttgtgg | |
| WTP211 | ccgaccggtgctcttcctcatcgccccgagcg | |
| WTP212 | caacgccgcccaccggtcggccaccatgg | pWT069 |
| WTP213 | cgtccgacatttaattcctcctgttagcccaaaaaacgggtatggagaaacagtagagag | |
| WTP214 | gaggaattaaatgtcggacgtcgaaaccacgc | |
| WTP215 | ccgaccggtgggcggcgttggcggc | |
| WTP216 | ggcgatccagcaccggtcggccaccatgg | pWT070 |
| WTP217 | gaagcgtcatttaattcctcctgttagcccaaaaaacgggtatggag | |
| WTP218 | gaggaattaaatgacgcttcgcaccccccg | |
| WTP219 | ccgaccggtgctggatcgccttcgagacggcg | |
| WTP220 | cccgctgagccaccggtcggccaccatgg | pWT071 |
| WTP221 | gttcggacaattaattcctcctgttagcccaaaaaacgggtatggag | |
| WTP222 | gaggaattaattgtccgaacaactcgacgccgtggatgc | |
| WTP223 | ccgaccggtggctcagcgggctgacatacgg | |
| WTP224 | ggaagcgggccaccggtcggccaccatgg | pWT072 |
| WTP225 | gttttggtcatttaattcctcctgttagcccaaaaaacgggtatggag | |
| WTP226 | ggaggaattaaatgaccaaaacggtccttgtcgtcga | |

| | | |
|---|---|---|
| WTP227 | ccgaccggtggcccgcttccttcatgtcgacttcg | |
| WTP228 | ggaagagggccaccggtcggccaccatgg | pWT073 |
| WTP229 | ggtcggccatttaattcctcctgttagcccaaaaaacgggtatggagaaacagtagagag | |
| WTP230 | gaggaattaaatggccgaccacgacgacc | |
| WTP231 | ccgaccggtggccctcttcctcgtccggct | |
| WTP240 | gaaggcggcccaccggtcggccaccatgg | pWT076 |
| WTP241 | aaatgcgcaattaattcctcctgttagcccaaaaaacgggtatggag | |
| WTP242 | gaggaattaattgcgcatttcgggaaccttgaacc | |
| WTP243 | ccgaccggtgggccgccttctggcgcac | |
| WTP001 | aggcaagggctaaggatccgcggccgctga | pWT001 |
| WTP002 | cggatccttagcccttgccttccgaggcggc | |
| WTP106 | aggcaagggctaaggatccgcggccgct | pWT037 |
| WTP107 | tgctcaccatgctagcgccctgaaaatacaggttttcactagt | |
| WTP108 | gggcgctagcatggtgagcaagggcgaggag | |
| WTP109 | cggatccttagcccttgccttccgaggcg | |
| WTP114 | gctgtacaagtaaggatccgcggccgctgagcaa | pWT039 |
| WTP115 | tgctcaccatgctagcgccctgaaaatacaggttttcactagt | |
| WTP116 | gggcgctagcatggtgagcaagggcgaggag | |
| WTP117 | cggatccttacttgtacagctcgtccatgccgagag | |

| | | |
|---|---|---|
| WTP268 | aggcaagggctaaggatccgcggccgctgagcaataactagc | pWT086 |
| WTP269 | cggcgcccaaggtggccgaccggtgcttgtacagctcgtccatgccgag | |
| WTP270 | gctgtacaagcaccggtcggccaccttgggcgccgtcgagactgcc | |
| WTP271 | cggatccttagcccttgccttccgaggcg | |
| WTP449 | ggatcagcgctaaggatccgcggccgctga | pWT141 |
| WTP450 | gcggatccttagcgctgatccagggcctggaa | |
| WTP451 | acgttccagctaaggatccgcggccgctgag | pWT142 |
| WTP452 | gcggatccttagctggaacgttcgtggcgtt | |
| WTP455 | gtcggccacccaggaactggtcgaccgcat | pWT144 |
| WTP456 | ccagttcctgggtggccgaccggtgcttgt | |
| WTP459 | ggatcagcgccaggaactggtcgaccgcat | pWT146 |
| WTP460 | ccagttcctggcgctgatccagggcctggaac | |
| WTP534 | gaacgttccagctaaggatccgcggccgctgagca | pWT170 |
| WTP535 | gcggatccttagctggaacgttcgtggcgtt | |
| WTP503 | tcatcatcacatggtgagcaagggcgag | pWT154 |
| WTP504 | tcgggctttgctacttcttcttgaacaggcccgagaacatcg | |
| WTP505 | gaagaagtagcaaagcccgaaaggaagctg | |
| WTP506 | tgctcaccatgtgatgatgatgatgatggctgct | |
| WTP492 | tcatcatcacatgggcagacacgggggg | pWT156 |

| | | |
|---|---|---|
| WTP493 | tcgggctttgttacttgtacagctcgtccatgccgccgg | |
| WTP490 | gtacaagtaacaaagcccgaaaggaagctgagttgg | |
| WTP494 | gtctgcccatgtgatgatgatgatgatggctgctgccc | |
| WTP532 | tcatcatcacaaggccgaggtcgcccatcg | pWT169 |
| WTP533 | gacctcggccttgtgatgatgatgatgatggc | |
| WTP552 | ctcggggacgcggcgcctaacaaagcccgaaaggaagctg | pWT187 |
| WTP553 | tcctcgcccttgctcaccatgtgatgatgatgatgatggctgctgc | |
| WTP554 | gccatcatcatcatcatcacatggtgagcaagggcgag | |
| WTP555 | cagcttcctttcgggctttgttaggcgccgcgtccc | |
| WTP556 | ccgtcggcgtgatggactaacaaagcccgaaaggaagct | pWT189 |
| WTP557 | acctgatgacgcggtttcatgtgatgatgatgatgatggctgctgcc | |
| WTP558 | gccatcatcatcatcatcacatgaaaccgcgtcatcaggt | |
| WTP559 | cagcttcctttcgggctttgttagtccatcacgccgacgg | |
| WTP560 | ccgtcggcgtgatggactaacaaagcccgaaaggaagct | pWT150 |
| WTP561 | tcctcgcccttgctcaccatgtgatgatgatgatgatggctgctgc | |
| WTP562 | gccatcatcatcatcatcacatggtgagcaag | |
| WTP563 | acctgatgacgcggtttcatggtggccgaccggtg | |
| WTP564 | acaagcaccggtcggccaccatgaaaccgcgtcatcaggt | |
| WTP565 | cagcttcctttcgggctttgttagtccatcac | |

| | | |
|---|---|---|
| WTP566 | gctgtacaagtaacaaagcccgaaaggaagctgag | pWT190 |
| WTP567 | ccgaccggtggtccatcacgccgacgg | |
| WTP568 | cgtgatggaccaccggtcggcc | |
| WTP569 | ggctttgttacttgtacagctcgtccatgccg | |
| WTP574 | agacgaccatatggtgagcaagggcgag | pWT205 |
| WTP575 | ccgaccggtgcttgtacagctcgtccatgcc | |
| WTP576 | gctgtacaagcaccggtcggccac | |
| WTP577 | tgctcaccatatggtcgtctccccaaaact | |
| WTP570 | gaaggcaagggctaaaaaacgggccccccctcga | pWT206 |
| WTP571 | ccgtttttttagcccttgccttccgaggcgg | |
| WTP578 | gtcggccaccatgtccgatcag | pWT207 |
| WTP579 | gccaagcttcttaggcgccgcgtccc | |
| WTP580 | cggcgcctaagaagcttggctgttttggcggatg | |
| WTP581 | gatcggacatggtggccgaccggtg | |
| WTP582 | gtcggccaccatggctatgaagctcgccgtca | pWT025 |
| WTP583 | caagcttcttatcagtagatcagttcgtcgtcgg | |
| WTP584 | tgatctactgataagaagcttggctgttttggcg | |
| WTP585 | tcatagccatggtggccgaccggtg | |
| WTP586 | cgaggaaacgatggtgagcaagggcgag | pWT195 |

| | | |
|---|---|---|
| WTP587 | ggcccgttttctacttcttcttgaacaggcccg | |
| WTP588 | gaagaagtagaaaacgggcccccccctcg | |
| WTP589 | tgctcaccatcgtttcctcgcatcgtgg | |
| WTP590 | gtcggccaccatggctatgaagctcgccg | pWT200 |
| WTP591 | ccgttttctagtagatcagttcgtcgtcggc | |
| WTP592 | actgatctactagaaaacgggcccccc | |
| WTP593 | tcatagccatggtggccgaccggtg | |
| WTP598 | gctgtacaagtaacaaagcccgaaaggaag | pWT209 |
| WTP599 | ggctttgttacttgtacagctcgtccatgc | |
| WTP693 | tccaggccctggatcagcgctaagaagctt | pWT246 |
| WTP694 | gccaaaacagccaagcttcttagcgctgatcc | |
| WTP689 | ggaaacgcatatggtgagcaagggcgagg | pWT243 |
| WTP690 | ccgctctagactactcttcgtcgctcacatcgg | |
| WTP691 | cgaagagtagtctagagcggccattcactggcc | |
| WTP692 | tgctcaccatatgcgtttcctcgcatcgtg | |

**Supplementary Table 6. Constructions of His-tagged proteins used in this study.**

| Protein | Synonyms | Mass (kDa) | Isoelectric point |
|---|---|---|---|
| PodJ_N | $PodJ_{1-601}$ | 68 | 4.93 |
| SpmX(ΔTM) | $SpmX_{1-350}$ | 38.8 | 4.97 |
| YFP | | 30.2 | 5.45 |
| YFP-PodJ_N | YFP-$PodJ_{1-601}$ | 95.5 | 5.06 |
| YFP-$PodJ_{CC1-3}$ | YFP-$PodJ_{1-249}$ | 57.5 | 5.32 |
| YFP-$PodJ_{CC4-6}$ | YFP-$PodJ_{250-430}$ | 50.3 | 5.67 |
| YFP-$PodJ_{IDR}$ | YFP-$PodJ_{431-601}$ | 49.2 | 4.87 |
| YFP-PodJ_N(ΔCC1-3) | YFP-$PodJ_{250-601}$ | 68.8 | 5.07 |
| YFP-PodJ_N(ΔCC4-6) | YFP-$PodJ_{1-249, 431-601}$ | 76 | 4.92 |
| YFP-PodJ_N(ΔIDR) | YFP-$PodJ_{1-430}$ | 77.1 | 5.44 |
| mCherry | | 27.9 | 5.8 |
| SpmX(ΔTM)-mCherry | $SpmX_{1-350}$-mCherry | 66.1 | 5.22 |
| PleC(ΔTM)-mCherry | $PleC_{307-842}$-mCherry | 87.7 | 5.8 |
| mCherry-CpaE | | 83.6 | 5.21 |

## Supplementary references


1. Holehouse, A. S., Das, R. K., Ahad, J. N., Richardson, M. O. G., Pappu, R. V. CIDER: resources to analyze sequence-ensemble relationships of intrinsically disordered proteins. *Biophys. J.* **112**, 16-21 (2017).
2. Zhang, H., et al. Liquid-liquid phase separation in biology: mechanisms, physiological functions and human diseases. *Sci China Life Sci* **63**, 953-985 (2020).
3. Oshidari, R., et al. DNA repair by Rad52 liquid droplets. *Nat Commun* **11**, 695 (2020).
4. Lindner, A. B., Madden, R., Dernarez, A., Stewart, E. J., Taddei, F. Asymmetric segregation of protein aggregates is associated with cellular aging and rejuvenation. *Pro. Natl Acad. Sci. USA* **105**, 3076-3081 (2008).
5. Taylor, N. O., Wei, M.-T., Stone, H. A., Brangwynne, C. P. Quantifying dynamics in phase-separated condensates using fluorescence recovery after photobleaching. *Biophys. J.* **117**, 1285-1300 (2019).
6. Viollier, P. H., Sternheim, N., Shapiro, L. Identification of a localization factor for the polar positioning of bacterial structural and regulatory proteins. *Pro. Natl Acad. Sci. USA* **99**, 13831-13836 (2002).
7. Bowman, G. R., et al. A polymeric protein anchors the chromosomal origin/ParB complex at a bacterial cell pole. *Cell* **134**, 945-955 (2008).

8. Huitema, E., Pritchard, S., Matteson, D., Radhakrishnan, S. K., Viollier, P. H. Bacterial birth scar proteins mark future flagellum assembly site. *Cell* **124**, 1025-1037 (2006).
9. Radhakrishnan, S. K., Thanbichler, M., Viollier, P. H. The dynamic interplay between a cell fate determinant and a lysozyme homolog drives the asymmetric division cycle of *Caulobacter crescentus*. *Gene Dev* **22**, 212-225 (2008).
