# Supplementary material for: Phase separation modulates the assembly and dynamics of a polarity-related scaffold-signaling hub
Source: Nat Commun. 2022 Nov 23;13:7181. doi: 10.1038/s41467-022-35000-2 (PMC9684454; doi:10.1038/s41467-022-35000-2)
Supplement: Supplementary file 2 — Description of Additional Supplementary Files [file 41467_2022_35000_MOESM2_ESM.pdf]

Description of Additional Supplementary Files

File Name: Supplementary Video 1

Description: The fusion process of two separated YFP-PodJ_N droplets. Corresponding to Fig. 2b.

File Name: Supplementary Video 2

Description: The rapid fluorescence recovery of YFP-PodJ_N droplets after photobleaching. Corresponding to Fig. 2c.

File Name: Supplementary Video 3

Description: The interfacial interaction between YFP-PodJ_N and SpmX(ΔTM)-mCherry. Corresponding to Fig. 5d.
