# Supplementary material for: Phase separation modulates the assembly and dynamics of a polarity-related scaffold-signaling hub
Source: Nat Commun. 2022 Nov 23;13:7181. doi: 10.1038/s41467-022-35000-2 (PMC9684454; doi:10.1038/s41467-022-35000-2)
Supplement: Supplementary file 6 — Reporting Summary [file 41467_2022_35000_MOESM6_ESM.pdf]

nature portfolio

Corresponding author(s): Wei Zhao

Last updated by author(s): Nov 1, 2022

Reporting Summary

Nature Portfolio wishes to improve the reproducibility of the work that we publish. This form provides structure for consistency and transparency in reporting. For further information on Nature Portfolio policies, see our Editorial Policies and the Editorial Policy Checklist.

Statistics

For all statistical analyses, confirm that the following items are present in the figure legend, table legend, main text, or Methods section.

n/a	Confirmed	
☐	☒	The exact sample size (n) for each experimental group/condition, given as a discrete number and unit of measurement
☐	☒	A statement on whether measurements were taken from distinct samples or whether the same sample was measured repeatedly
☐	☒	The statistical test(s) used AND whether they are one- or two-sided<br>*Only common tests should be described solely by name; describe more complex techniques in the Methods section.*
☒	☐	A description of all covariates tested
☒	☐	A description of any assumptions or corrections, such as tests of normality and adjustment for multiple comparisons
☐	☒	A full description of the statistical parameters including central tendency (e.g. means) or other basic estimates (e.g. regression coefficient) AND variation (e.g. standard deviation) or associated estimates of uncertainty (e.g. confidence intervals)
☐	☒	For null hypothesis testing, the test statistic (e.g. F, t, r) with confidence intervals, effect sizes, degrees of freedom and P value noted<br>*Give P values as exact values whenever suitable.*
☒	☐	For Bayesian analysis, information on the choice of priors and Markov chain Monte Carlo settings
☒	☐	For hierarchical and complex designs, identification of the appropriate level for tests and full reporting of outcomes
☒	☐	Estimates of effect sizes (e.g. Cohen's d, Pearson's r), indicating how they were calculated

Our web collection on statistics for biologists contains articles on many of the points above.

Software and code

Policy information about availability of computer code

Data collection	Cell imaging: Nikon Eclipse Ti2-E inverted fluorescence microscope<br>Confocal imaging and FRAP: Nikon A1R+ confocal laser scanning microscope<br>TEM: FEI Tecnai Spirit BioTWIN electron microscopy<br>SDS-PAGE: Microtek Bio-5000 Plus scanner
Data analysis	ImageJ v1.53f51, 3Dscript and MTrackJ (Particle tracking and intensity analysis);<br>GraphPad Prism v5.0 (Graph plotting, statistical analysis);<br>MicrobeJ v5.13 (Kymographs and protein subcellular analysis);<br>Metadisorder MD2, SPOT-Disorder2, Cspritz v1.2, IUPred2, TOPCONS, HHpred v2.08, CIDER and RADAR (Bioinformatic analysis)

For manuscripts utilizing custom algorithms or software that are central to the research but not yet described in published literature, software must be made available to editors and reviewers. We strongly encourage code deposition in a community repository (e.g. GitHub). See the Nature Portfolio guidelines for submitting code & software for further information.

Data

Policy information about availability of data

All manuscripts must include a data availability statement. This statement should provide the following information, where applicable:

- Accession codes, unique identifiers, or web links for publicly available datasets
- A description of any restrictions on data availability
- For clinical datasets or third party data, please ensure that the statement adheres to our policy

Data supporting the findings of this study are available within the main manuscript, the Supplementary Information, and the Supplementary Videos. Relevant raw

microscopy images are available from the corresponding author upon reasonable request. Source data are provided with this paper.

Field-specific reporting

Please select the one below that is the best fit for your research. If you are not sure, read the appropriate sections before making your selection.

[x] Life sciences [ ] Behavioural & social sciences [ ] Ecological, evolutionary & environmental sciences

For a reference copy of the document with all sections, see nature.com/documents/nr-reporting-summary-flat.pdf

Life sciences study design

All studies must disclose on these points even when the disclosure is negative.

Sample size	Sample sizes were not predetermined. We generally used sample sizes of 3 or more biological replicates, which can provide necessary statistical support. Sample sizes are indicated in the manuscript.
Data exclusions	No data was excluded from analysis.
Replication	All experiments were repeated at least three times independently.
Randomization	Not relevant. Samples were not allocated into separate experimental groups. All experiments were performed with appropriate negative and positive controls in keeping with the standards of the field.
Blinding	The samples were grouped blinded during data collection and analysis.

Reporting for specific materials, systems and methods

We require information from authors about some types of materials, experimental systems and methods used in many studies. Here, indicate whether each material, system or method listed is relevant to your study. If you are not sure if a list item applies to your research, read the appropriate section before selecting a response.

Materials & experimental systems

n/a	Involved in the study	
[x]	[ ]	Antibodies
[x]	[ ]	Eukaryotic cell lines
[x]	[ ]	Palaeontology and archaeology
[x]	[ ]	Animals and other organisms
[x]	[ ]	Human research participants
[x]	[ ]	Clinical data
[x]	[ ]	Dual use research of concern

Methods

n/a	Involved in the study	
[x]	[ ]	ChIP-seq
[x]	[ ]	Flow cytometry
[x]	[ ]	MRI-based neuroimaging
